# Digital Interventions for Cognitive Dysfunction in Patients With Stroke: Systematic Review and Meta-Analysis

**DOI:** 10.2196/73687

**Published:** 2025-07-24

**Authors:** Chen Wang, Min Liu

**Affiliations:** 1 School of Rehabilitation Medicine, Shandong University of Traditional Chinese Medicine, Jinan, China; 2 Department of Rehabilitation Medicine, Shandong Provincial Third Hospital, 11 Wuyingshan Middle Road, Jinan, 250031, China, 86 18660126767

**Keywords:** stroke, cognitive impairment, virtual reality, computer-assisted therapy, robotics, cognitive training

## Abstract

**Background:**

In recent years, digital technologies have shown possibilities for improving cognitive function after stroke, but their effectiveness and treatment options vary, the optimal treatment remains unclear, and the current evidence is somewhat contradictory.

**Objective:**

This study aimed to evaluate the efficacy of various digital interventions in improving poststroke cognitive function and provide evidence-based support for clinical decision-making.

**Methods:**

A systematic search was conducted across PubMed, Web of Science, Cochrane Library, Scopus, Embase, and CNKI databases from their inception to January 2025, with no restrictions on language or publication year. Randomized controlled trials evaluating digital interventions (eg, virtual reality [VR], computer-assisted cognitive therapy [CACT], and robot-assisted therapy [RAT]) for poststroke cognitive impairment in adults (aged≥18 y) were included. Eligible studies reported outcomes measured by the Montreal Cognitive Assessment (MoCA) or the Mini-Mental State Examination (MMSE), with cognitive improvement quantified through pre- to postintervention scores. Multiple researchers independently extracted data. Network meta-analysis was performed using R software, incorporating consistency or inconsistency models (based on Deviance Information Criterion differences), random-effects models, and *I*² statistics to assess heterogeneity. Sources of heterogeneity were analyzed through sensitivity analyses, subgroup analyses, and meta-regression. Intervention efficacy was ranked using Surface Under the Cumulative Ranking Curve (SUCRA) values. Robustness and consistency were validated via Egger test, sensitivity analyses, and node-splitting methods. Evidence quality was assessed using the Grading of Recommendations Assessment, Development, and Evaluation framework.

**Results:**

A total of 2128 articles were retrieved, with 27 meeting the inclusion criteria. Compared to conventional rehabilitation or care (C), CACT demonstrated significant superiority in MoCA scores (mean difference [MD]=3.03, 95% CI 1.69 to 4.38; SUCRA=91.53%); while cognitive training (CCT) demonstrated no statistical difference (MD=0.70, 95% CI −0.88 to 2.28). The ordering is CACT>VR>RAT>CCT. For MMSE scores, RAT ranked highest in efficacy (MD=5.99, 95% CI 3.20 to 8.79; SUCRA=99.44%); whereas both VR (MD=1.34, 95% CI −0.94 to 3.62) and CCT (MD=1.12, 95% CI −1.46 to 3.69) showed no significant improvement. The ordering is RAT>CACT>CCT>VR.

**Conclusions:**

Digital therapies are effective in improving cognitive functioning in patients post stroke. CACT showed superior efficacy on the MoCA (emphasizing executive functioning), while RAT had the highest efficacy in the MMSE (focusing on basic cognition), suggesting different domain-specific effects. However, caution is warranted due to the heterogeneity of the included studies, risk of bias, and limited sample sizes in some studies. Future research should focus on optimizing intervention protocols, integrating neuromodulation or traditional rehabilitation techniques, and exploring cost-effective clinical implementation strategies.

## Introduction

Poststroke cognitive impairment (PSCI) is a debilitating complication that affects functional recovery and quality of life in stroke survivors, with a prevalence exceeding 70% [1]. Cognitive deficits typically span multiple domains [2], including attention, memory, executive function, and spatial orientation. These deficits not only compromise patients’ independence in daily activities but also contribute to psychological comorbidities such as depression and anxiety through impaired self-efficacy [3]. Critically, untreated PSCI may progress to dementia [4], underscoring the urgent need for effective rehabilitation strategies. Conventional cognitive rehabilitation methods (eg, paper-based exercises, occupational therapy), while widely used in clinical practice, are constrained by standardization deficiencies, low personalization, and repetitive task paradigms lacking ecological validity. These challenges are particularly pronounced in resource-constrained health care settings, where long-term accessibility is hindered by workforce shortages and inequitable resource allocation. Such limitations highlight the pressing demand for scalable, adaptive interventions that align with individual patient profiles and cognitive domains.

Given the limitations of conventional approaches, digital therapies such as computer-assisted cognitive therapy (CACT), virtual reality (VR), and robot-assisted therapy (RAT) have emerged as promising alternatives. VR leverages immersive environments to integrate multisensory stimulation with task-oriented training [5], demonstrating unique advantages in enhancing visuospatial and executive functions [6]. Key components of VR, such as multimodal feedback (visual, auditory, and tactile), create comprehensive human-machine interactions that promote neuroplasticity [7]. CACT, driven by intelligent algorithms and online modules, enables personalized training with dynamic difficulty adjustments, showing significant potential in improving memory, attention, and information-processing speed [8]. Adaptive learning systems, a cornerstone of CACT, optimize task content and feedback mechanisms based on real-time performance data, forming closed-loop cognitive reinforcement pathways [9]. This precisely targeted intervention model not only enhances training efficiency but also facilitates the reorganization and compensation of functional brain networks through neurofeedback technology, thereby providing an evidence-based strategy to remodel impaired cognitive pathways [10]. In addition, with the continuous development of artificial intelligence (AI), AI-based cognitive intervention robots have been continuously applied to clinical practice [11]. It integrates the patient’s behavioral patterns through multimodal interaction and big data analysis, which not only enhances the fun and engagement of rehabilitation training but also provides the possibility of developing accurate rehabilitation programs [12].

Despite these advancements, there are significant contradictions in the available evidence: some studies concluded that VR is superior to conventional methods in spatial navigation training, and a meta-analysis by Lin et al [13] demonstrated that although the cognitive improvement effect was relatively low, the comparison with conventional therapies was still significant. While some studies showed no significant effect of VR in the area of cognitive improvement, for example, a study by Zhang et al [14], which summarized 23 randomized controlled trials (RCTs) by meta-analysis, found that VR had a significant effect on executive and visuospatial functions in stroke patients, whereas the improvement in overall cognitive function did not show statistically significant differences. These discrepancies may stem from methodological heterogeneity: the analysis by Lin et al [13] prioritized VR systems integrating task-specific executive challenges (eg, Reh@City), whereas the review by Zhang et al [14] included studies with nonstandardized VR protocols lacking adaptive difficulty or multisensory feedback. CACT may be advantageous in terms of improvements in executive and memory functions, but its significance compared with other digital therapies has been less well studied. In addition, there are issues such as the efficacy association among intervention dose, type of technique, and cognitive improvement, which remains unclear. RAT is predominantly applied in motor rehabilitation [15,16], with limited exploration in cognitive domains.

Despite the growing interest in digital therapies, current systematic evaluations and meta-analyses [14,17,18] have focused primarily on single technologies versus traditional rehabilitation, and there is a lack of network meta-analysis (NMA) between different digital interventions (eg, VR vs CACT vs RAT).

Unlike traditional meta-analyses, which are limited to comparing 2 interventions simultaneously, NMA allows for the simultaneous comparison of multiple therapies (eg, VR vs CACT vs RAT) through indirect evidence synthesis. This approach not only resolves the ambiguity of heterogeneous pairwise results but also produces clinically interpretable rankings (via Surface Under the Cumulative Ranking Curve [SUCRA] values) to identify the most effective interventions for different cognitive domains.

This NMA addresses these gaps by: (1) quantifying the relative efficacy of different digital interventions in improving PSCI, (2) identifying optimal intervention strategies for specific cognitive domains (eg, the Montreal Cognitive Assessment [MoCA] for executive function vs the Mini-Mental State Examination [MMSE] for global cognition), and (3) evaluating clinical feasibility and guiding future research directions for technology integration and protocol optimization.

The findings aim to provide clinicians with a decision-making framework for selecting optimal therapies based on individualized cognitive deficits, while guiding future research toward protocol standardization and mechanistic validation.

## Methods

### Protocol and Registration

This study is a systematic review and network meta-analysis. The protocol was registered on PROSPERO (CRD420251006601) and adheres to the guidelines outlined in the Cochrane Handbook for Systematic Reviews of Interventions. The PRISMA (Preferred Reporting Items for Systematic Reviews and Meta-Analyses) guidelines were rigorously followed to ensure methodological transparency and reporting quality (see the PRISMA checklist in Checklist 1).

### Data Sources and Search Strategy

In total, 6 databases—PubMed, Web of Science, Cochrane Library, Scopus, Embase, and CNKI—were systematically searched from their inception to January 2025. Two authors independently developed the search strategy to ensure methodological rigor. The search query was adapted for each database to account for differences in indexing systems, syntax, and terminology. For international databases (PubMed, Web of Science, Cochrane, Scopus, and Embase), we combined MeSH (Medical Subject Headings) and Emtree terms (eg, stroke, cognitive dysfunction, VR, and robotics) with free-text keywords (eg, PSCI, CACT, and RAT). For the CNKI database, the search strategy was translated into Chinese and optimized using discipline-specific terms (eg, 卒中后认知障碍, 虚拟现实, 计算机辅助认知训练) to align with its indexing framework. Boolean operators and proximity filters were adjusted per database requirements. No language or publication year restrictions were applied. The full search syntax for all databases, including adaptations for CNKI, is provided in Multimedia Appendix 1.

### Eligibility Criteria

Two authors independently screened and selected studies based on predefined inclusion and exclusion criteria. Discrepancies were resolved through discussion or consultation with a third reviewer. Detailed records of study selection, including methodological quality assessment and data extraction, were maintained throughout the process.

### Inclusion Criteria

Inclusion criteria were based on the PICOS (Population, Intervention, Control, Outcome, and Study Design) program. These are summarized in Table 1.

**Table 1.** Inclusion criteria.

PICOS elements	Inclusion criteria
Population	Adult patients (>18 years old) with a clinical diagnosis of stroke and poststroke cognitive impairment
Intervention	Digital therapy intervention group: virtual reality, computer-assisted cognitive therapy, robot-assisted therapy, conventional cognitive training
Comparator	Control group receiving traditional cognitive rehabilitation (eg, paper-and-pencil tasks, occupational therapy, or usual care) or digital cognitive intervention
Outcomes	Quantifying Cognitive Improvement Using the Montreal Cognitive Assessment or the Brief Mental State Examination
Study Design	Randomized controlled trial

### Exclusion Criteria

Exclusion criteria regarding population, intervention, control, outcome, and study design are shown in Table 2.

**Table 2.** Exclusion criteria.

PICOS elements	Exclusion criteria
Population	Patients who are comatose or unable to participate in cognitive interventions Groups with comorbidities of other neurodegenerative diseases (eg, Alzheimer disease) Minors
Intervention	Intervention deviation from preset numeric therapies (eg, pharmacologic interventions and nonnumeric therapies such as acupuncture)
Comparator	Control group with unconventional rehabilitation (eg, drug control and sham surgery) or nondigital cognitive interventions unrelated to this study (eg, neuromodulation techniques)
Outcomes	Endpoints are reported in nonquantitative forms (eg, qualitative descriptions) Missing raw data do not allow calculation of effect sizes
Study design	Conference abstracts, case reports, non-RCT^a^ study designs

a RCT: randomized controlled trial.

### Data Extraction and Quality Assessment

#### Data Extraction

Eligible studies were independently reviewed and extracted by 2 authors, with discrepancies resolved through consultation with a third reviewer. The following data were systematically collected: study title, publication year, authors, participant characteristics, intervention details, outcome measures, and quality assessment.

The methodological quality of included studies was evaluated using the revised Cochrane Risk of Bias tool. A total of 5 domains were assessed: risk of bias in randomization, risk of bias in intervention allocation, risk of bias due to missing data, risk of bias in outcome measurement, and risk of selective reporting bias. Studies were classified as (1) low risk: all domains rated “low risk” or (2) high risk: at least one domain rated “high risk.”

#### Data Analysis

We conducted a NMA using the “netmeta” and “gemtc” packages in R software. Treatment effects were estimated using mean differences (MDs) with 95% CI.

Two models (consistency and inconsistency) were compared based on Deviance Information Criterion (DIC) values. A DIC difference threshold of ≥5 was applied to select between models, as recommended in methodological guidelines for network meta-analyses [19,20]. This threshold reflects a balance between model parsimony and goodness-of-fit: differences<5 suggest negligible improvement in model fit when accounting for inconsistency, favoring the simpler consistency model. Conversely, differences≥5 indicate substantial divergence between direct and indirect evidence, necessitating the inconsistency model.

Heterogeneity across studies was quantified using the *I*² statistic, interpreted as follows: *I*²≤25% (low), 25%<*I*²≤50% (moderate), and *I*²>75% (high). To account for between-study variability, a random-effects model was applied.

Forest plots visualized effect sizes for interventions versus conventional rehabilitation, and network plots mapped connectivity between treatments.

Intervention efficacy was ranked using SUCRA values, where higher percentages denote superior effectiveness. Pairwise comparisons were synthesized in league tables, highlighting statistically significant differences (95% CI excluding zero).

#### Robustness and Statistical Validation

To ensure the reliability of the findings, we rigorously validated the convergence of the NMA by examining trace plots and density plots for stability. Global and local inconsistencies were assessed using the node-splitting method. A significance threshold of *P*<.05 was applied to identify inconsistencies between direct and indirect evidence, prompting the use of an inconsistency model to resolve discrepancies. Conversely, *P*≥.05 indicated consistency, allowing the application of a consistency model for final analysis.

Publication bias was assessed by Egger test and funnel plot symmetry assessment. Sensitivity analyses using the leave-one-out method assessed robustness by repeatedly excluding individual studies, and when there was a significant decrease in overall heterogeneity with the removal of a study, it indicated that this one study was a highly likely source of heterogeneity. Subgroup analyses and meta-regression further explored sources of heterogeneity (eg, intervention programs and population characteristics).

### Evidence Quality Assessment

The evidence quality for each outcome was systematically evaluated using the Evidence quality was assessed using the Grading of Recommendations Assessment, Development, and Evaluation (GRADE) framework. If significant deficiencies were identified in domains such as risk of bias, heterogeneity, indirectness, imprecision, or publication bias, the evidence quality was downgraded from high to lower levels (eg, moderate, low, or very low) accordingly.

## Results

### Overview

**Figure 1.**
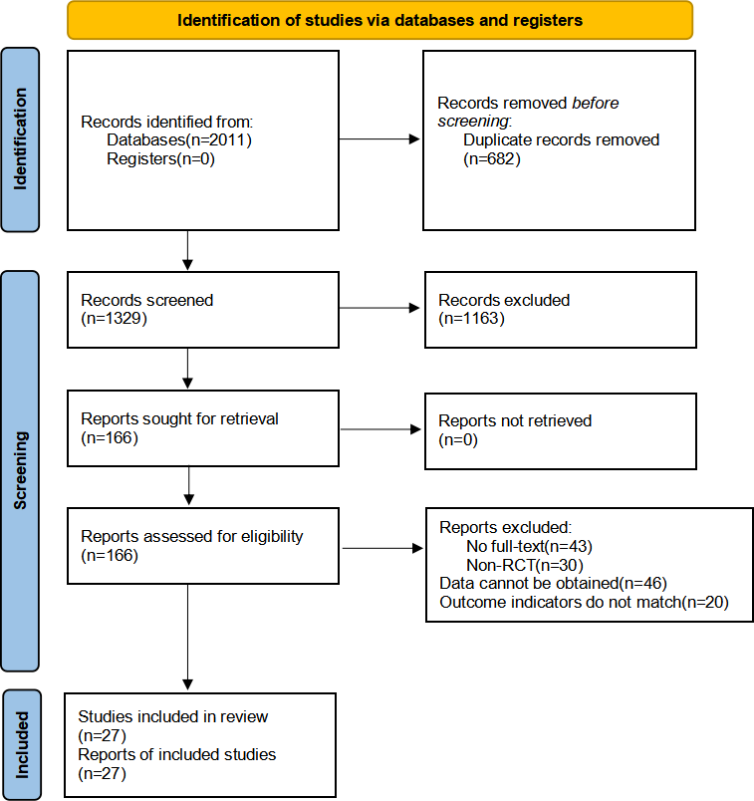
PRISMA (Preferred Reporting Items for Systematic Reviews and Meta-Analyses) flowchart of study selection for the systematic review and network meta-analysis of digital interventions in poststroke cognitive impairment.

A total of 2128 articles were retrieved from six databases: PubMed (n=226), Web of Science (n=411), Cochrane Library (n=374), Scopus (n=161), Embase (n=580), and CNKI (n=259). After removing 682 duplicates, 1329 articles underwent title or abstract screening, with 1163 excluded for irrelevance. Full-text review of 166 articles yielded 27 [21,22] eligible RCTs (Figure 1).

### Study Characteristics

A total of 27 studies with 1517 patients were included in the article, involving 5 different interventions, including VR technology, computer-assisted therapy, RAT, cognitive training, and conventional rehabilitation. There were 12 studies involving VR technology, 13 studies involving computer-assisted therapy, 4 studies involving RAT, and 8 studies involving cognitive training. The main characteristics of the included studies are shown in the table (Table 3).

**Table 3.** Characteristics of the included randomized controlled trials (RCTs) evaluating digital interventions for poststroke cognitive impairment (PSCI) in adults.

Study (year) and group		Disease duration, mean (SD)	Age (years), mean (SD)	Intervention	Intervention system or intervention modality	Sample size	Time	Measuring instrument
Faria et al [21] (2020)								
	TG^a^	45.93 (43.56) months	59.14 (11.81)	VR^b^	Reh@Cityv2.0	14	2 years	MoCA^c^
	CG^d^	21.33 (12.8) months	65.00 (6.20)	CCT^e^	Paper and pencil intervention	18	2 years	MoCA
Faria et al [23] (2018)								
	TG	24.90 (20.30) months	57.1 (11.0)	VR	Reh@Task	12	1 month	MoCA
	CG	41.10 (41.00) months	68.9 (9.8)	C^f^	Vocational and writing training	12	1 month	MoCA
Faria et al [24] (2016)								
	TG	7.00 (11.25) months	58.00 (3.83)	VR	Reh@City	9	4 weeks	MMSE^g^
	CG	4.00 (3.25) months	53.00 (5.75)	CCT	Cognitive training for time matching	9	4 weeks	MMSE
Baltaduoniene et al [25] (2019)								
	TG1	NOR^h^	73.67 (10.10)	VR	SeeMeR Brontes Processing	40	32 days	MoCA and MMSE
	TG2	NOR	73.67 (10.10)	CACT^i^	Fundamentals, memorization, problem solving, visuospatial	41	32 days	MoCA and MMSE
	CG	NOR	74.33 (10.27)	CCT	OT	40	32 days	MoCA and MMSE
Rogers et al [26] (2019)								
	TG	22.80 (14.80) days	64.30 (17.4)	VR	Elements mandates	10	4 weeks	MoCA
	CG	30.0 (15.90) days	64.60 (12.0)	C	OT and PT	11	4 weeks	MoCA
Kim et al [27] (2011)								
	TG	18.20 (11.30) days	66.50 (11.0)	VR	IREX systems	15	4 weeks	MMSE
	CG	24.00 (31.10) days	62.00 (15.8)	CACT	ComCog	13	4 weeks	MMSE
Oh et al [28] (2019)								
	TG	NOR	57.40 (12.2)	VR	Joystim	17	6 weeks	MoCA and MMSE
	CG	NOR	52.60 (10.7)	C	OT	14	6 weeks	MoCA and MMSE
Ozen et al [29] (2021)								
	TG	151.80 (98.40) days	62.00 (13.12)	CACT	Rejoyce	15	20 days	MoCA and MMSE
	CG	145.80 (86.10) days	62.00 (13.12)	C	OT	15	20 days	MoCA and MMSE
Soni et al [30] (2025)								
	TG	NOR	51.80 (6.25)	CACT	NOR	25	4 weeks	MoCA
	CG	NOR	49.40 (6.38)	C	Paper and pencil–based home training tasks	25	4 weeks	MoCA
Marcela et al [31] (2023)								
	TG	126.00 (15.58) days	59.36	VR	OculusQuest2	25	4 weeks	MoCA
	CG	128.00 (14.63) days	59.36	C		25	4 weeks	MoCA
Akinci et al [32] (2024)								
	TG	74.18 (33.77) days	59.18 (8.89)	VR	Lokomat	17	6 weeks	MoCA
	CG	66.71 (34.13) days	59.12 (13.22)	CCT	Paper and pencil intervention	17	6 weeks	MoCA
Jiang et al [33] (2016)								
	TG	40.56 (18.88) days	56.18 (11.86)	CACT	RehaCom	51	3 weeks	MoCA and MMSE
	CG	40.27 (19.17) days	59.56 (10.10)	C	Traditional rehabilitation	49	3 weeks	MoCA and MMSE
Wilson et al [34] (2021)								
	TG	137.50 (152.40) days	69.90 (13.8)	VR	Elements	10	8 weeks	MoCA
	CG	107.40 (56.40) days	77.30 (8.9)	C	GRASP	7	8 weeks	MoCA
Taravati et al [35] (2021)								
	TG	10.94 (8.02) months	50.94 (17.20)	RAT^k^	Reo Go-Motor rehabilitation system	17	4 weeks	MoCA
	CG	12.65 (8.42) months	55.75 (11.61)	C	Routine rehabilitation training	20	4 weeks	MoCA
Adomaviien et al [36] (2019)								
	TG	8.69 (4.27) weeks	66.00 (2.37)	VR	Kinect OR Armeo	25	10 days	MMSE
	CG	8.69 (4.27) weeks	62.00 (1.33)	C	NOR	25	10 days	MMSE
Liu et al [37] (2018)								
	TG	NOR	61.50 (12.34)	CACT	NOR	62	4 weeks	MoCA
	CG	NOR	62.35 (10.34)	C	NOR	66	4 weeks	MoCA
De Luca et al [38] (2017)								
	TG	3 (1) months	43.90 (16.60)	CACT	Istitutodi Ricercae Curaa Carattere Scientifico Neurolesi	20	8 weeks	MMSE
	CG	4 (1) months	42.10 (17.70)	C	Traditional rehabilitation	15	8 weeks	MMSE
Zucchella et al [39] (2014)								
	TG	NOR	64.00 (13.33)	CACT	Unapalestraperlamente	42	4 weeks	MMSE
	CG	NOR	70.00 (10.37)	C	Routine rehabilitation training	45	4 weeks	MMSE
Choi et al [40] (2014)								
	TG	20.20 (14.10) days	64.30 (10.30)	VR	business game	10	4 weeks	MMSE
	CG	23.67 (20.70) days	64.70 (11.30)	C	OT	10	4 weeks	MMSE
Meng et al [41] (2022)								
	TG	8.38 (2.05) weeks	56.25 (5.38)	CACT	DKYYZ	40	3 months	MMSE
	CG	8.8 (2.63) weeks	57.40 (6.04)	CCT	NOR	40	3 months	MMSE
Luo et al [42] (2020)								
	TG1	NOR	NOR	CACT	CAARS	30	12 weeks	MoCA
	TG2	NOR	NOR	CCT	Artificial Cognitive Function Training	30	12 weeks	MoCA
	CG	NOR	NOR	C	NOR	30	12 weeks	MoCA
Zhang et al [43] (2020)								
	TG	3.20 (4.70) years	71.50 (5.30)	CACT	NOR	37	6 weeks	MMSE
	CG	3.50 (4.30) years	71.60 (5.70)	CCT	Routine rehabilitation training	37	6 weeks	MMSE
Zhang and Tian [44] (2020)								
	TG	NOR	60.72 (0.88)	CACT	NOR	30	NOR	MoCA
	CG	NOR	59.62 (0.85)	C	Routine rehabilitation training	30	NOR	MoCA
Su et al [45] (2022)								
	TG	27.13 (3.88) days	64.97 (4.88)	RAT	Upper Extremity Rehabilitation Robotic Training	30	4 weeks	MoCA and MMSE
	CG	26.73 (4.08) days	65.53 (5.46)	CCT	Routine rehabilitation training	30	4 weeks	MoCA and MMSE
Xu et al [46] (2023)								
	TG	2.61 (1.32) months	51.66 (7.15)	RAT	ReFle ×100	32	3 months	MMSE
	CG	3.09 (1.21) months	52.94 (6.27)	C	Schuell stimulation	32	3 months	MMSE
Lu et al [22] (2022)								
	TG	5.12 (0.65) months	58.65 (2.16)	RAT	Lokomat Gait Assessment and Training and Integrated Upper Limb Training	48	1 month	MoCA
	CG	5.01 (0.63) months	58.61 (2.13)	C	Traditional rehabilitation	48	1 month	MoCA
Yao et al [47] (2021)								
	TG	6.20 (0.90) months	68.60 (6.20)	CACT	Computerized Cognitive Function Systems Training	40	3 weeks	MoCA and MMSE
	CG	5.90 (1.50) months	67.90 (6.50)	C	Routine rehabilitation training	40	3 weeks	MoCA and MMSE

a TG: treatment group.

b VR: virtual reality.

c MoCA: Montreal Cognitive Assessment Scale.

d CG: control group.

e CCT: cognitive training.

f C: conventional therapy.

g MMSE: Minimum Mental State Examination.

h NOR: not otherwise reported.

i CACT: computer-assisted cognitive therapy.

j OT: occupational therapy.

k RAT: robot-assisted therapy.

### Risk of Bias

**Figure 2.**
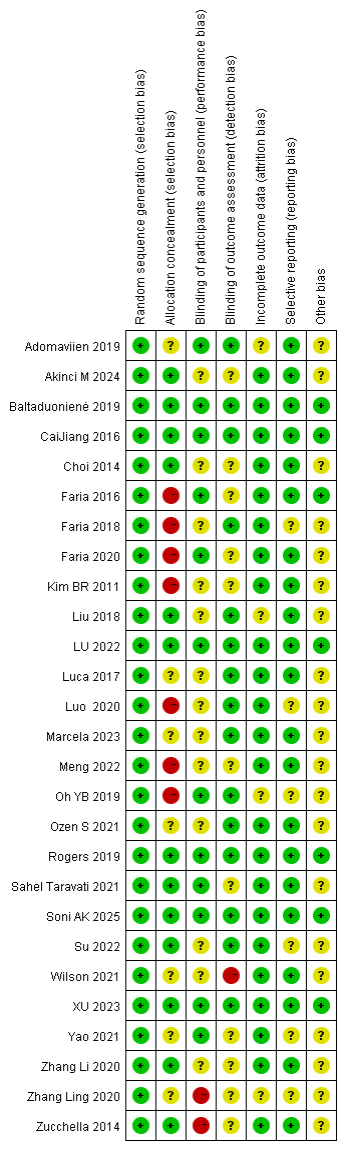
Risk-of-bias summary [21-47].

Twenty-seven RCTs were included in the analysis. Based on the revised Cochrane Risk of Bias tool, 10 studies were rated as high risk, 6 as low risk, and the remaining 11 as moderate risk (some concerns). Analysis revealed that some studies exhibited deficiencies in blinding and allocation concealment, which may introduce bias into the outcomes. However, outcome measurement and reporting generally demonstrated low risk of bias across all studies. A detailed risk of bias assessment is presented in Figure 2 [21-47].

### Net Meta-Analysis

#### Overview

The study begins by applying the DIC value to compare the 2 models (Consistent and Inconsistent models). For the MoCA as an outcome indicator, the DIC value was 64.15 for the consistent model and 66.64 for the inconsistent model; for the MMSE as an outcome indicator, the DIC value was 60.04 for the consistent model and 59.77 for the inconsistent model, and both comparisons revealed that the difference in the DIC value was less than 5, so the two were analyzed by applying the consistent model. The analysis results are as follows.

#### Primary Outcomes

**Figure 3.**
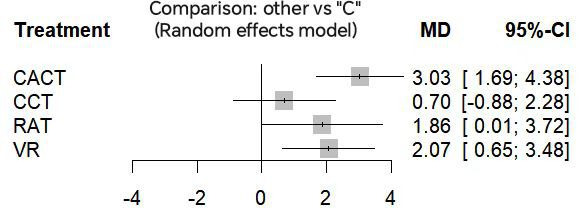
Forest plot for the Montreal Cognitive Assessment. C: conventional rehabilitation therapy; CACT: computer-assisted cognitive therapy; CCT: cognitive training; MD: mean difference; RAT: robot-assisted therapy; VR: virtual reality.

On the MoCA, compared to conventional therapies, CACT showed the greatest improvement (MD=3.03, 95% CI 1.69 to 4.38; SUCRA=91.53%). VR ranked second (MD=2.07, 95% CI 0.65 to 3.48; SUCRA=66.53%). RAT ranked second (MD=1.86, 95% CI 0.01 to 3.72; SUCRA=60.33%). Cognitive training (CCT) showed no significant difference compared to conventional therapy (Figure 3 and Table 4).

**Table 4.** League table for the Montreal Cognitive Assessment (MoCA) showing the mean differences in MoCA scores and their 95% CIs among the interventions.

Treatment	C^a^	CACT^b^	CCT^c^	RAT^d^	VR^e^
C	—^f^	—	—	—	—
CACT	−3.02 (−4.4 to −1.35)	—	—	—	—
CCT	−0.68 (−2.43 to 1.24)	2.33 (0.32 to 4.32)	—	—	—
RAT	−1.83 (−3.99 to 0.38)	1.19 (−1.45 to 3.65)	−1.15 (−3.58 to 1.15)	—	—
VR	−2.02 (−3.61 to −0.44)	0.99 (−1.03 to 2.84)	−1.33 (−3.15 to 0.29)	−0.18 (−2.70 to 2.27)	—

a C: conventional therapy.

b CACT: Computer-assisted cognitive therapy.

c CCT: cognitive training.

d RAT: robot-assisted therapy.

e VR: Virtual reality.

f Not applicable.

**Figure 4.**
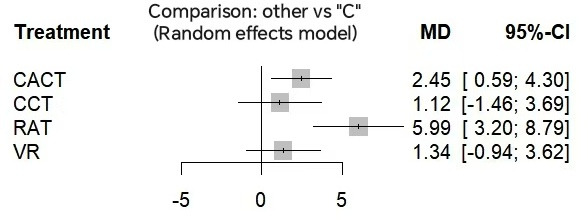
Forest plot for the Mini-Mental State Examination. C: conventional rehabilitation therapy; CACT: computer-assisted cognitive therapy; CCT: cognitive training; MD: mean difference; RAT: robot-assisted therapy; VR: virtual reality.

**Figure 5.**
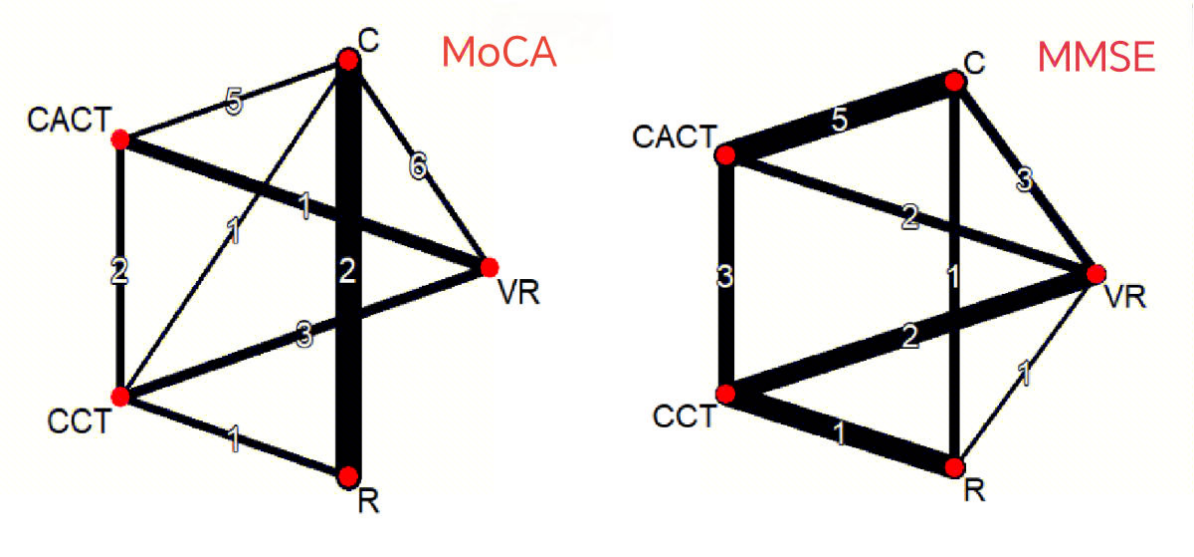
Network evidence diagram of included studies. C: conventional rehabilitation therapy; CACT: computer-assisted cognitive therapy; CCT: cognitive training; MMSE: Mini-Mental State Examination; MoCA: Montreal Cognitive Assessment.

On the MMSE, compared to conventional therapies, RAT was most effective (MD=5.99, 95% CI 3.20 to 8.79; SUCRA=99.44%). CACT ranked second (MD=2.45, 95% CI 0.59 to 4.30; SUCRA=67.8%). VR and CCT showed no significant benefits (Figure 4 and Table 5). The mesh of the MoCA and the MMSE is shown in Figure 5.

**Table 5.** League table for the Mini-Mental State Examination (MMSE) showing mean differences in MMSE scores and their 95% CIs among the interventions.

Treatment	C^a^	CACT^b^	CCT^c^	RAT^d^	VR^e^
C	—^f^	—	—	—	—
CACT	−2.49 (−4.37 to −0.41)	—	—	—	—
CCT	−1.19 (−3.78 to 1.78)	1.29 (−1.03 to 3.80)	—	—	—
RAT	−6.06 (−8.94 to −2.87)	−3.56 (−6.69 to −0.37)	−4.85 (−7.99 to −1.81)	—	—
VR	−1.36 (−3.68 to 1.15)	1.12 (−1.33 to 3.59)	−0.17 (−2.91 to 2.41)	4.68 (1.61 to 7.69)	—

a C: conventional therapy.

b CACT: Computer-assisted cognitive therapy.

c CCT: cognitive training.

d RAT: robot-assisted therapy.

e VR: Virtual reality.

f Not applicable.

#### Intervention Rankings

**Figure 6.**
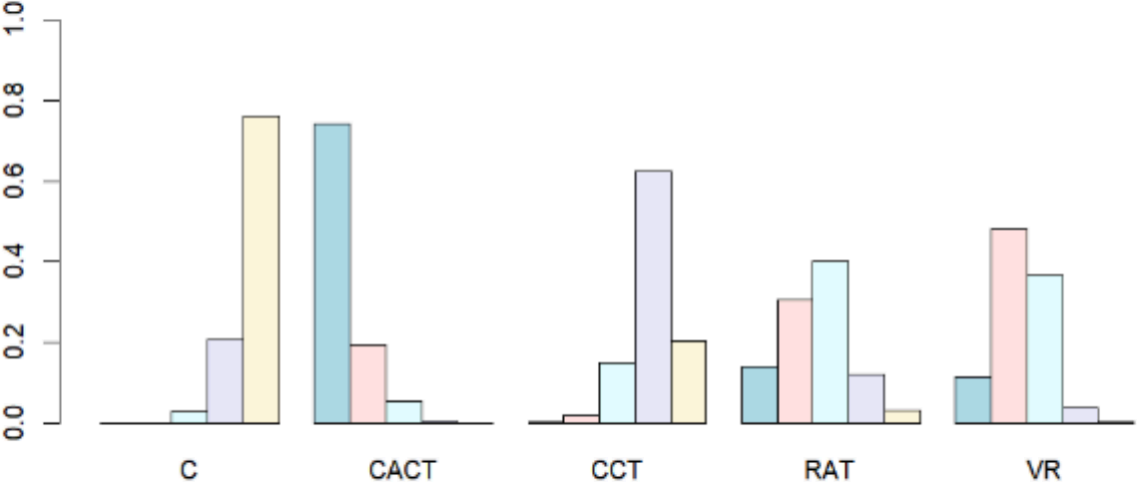
The Montreal Cognitive Assessment’s inclusion study stacking chart. C: conventional rehabilitation therapy; CACT: computer-assisted cognitive therapy; CCT: cognitive training; RAT: robot-assisted therapy; VR: virtual reality.

**Figure 7.**
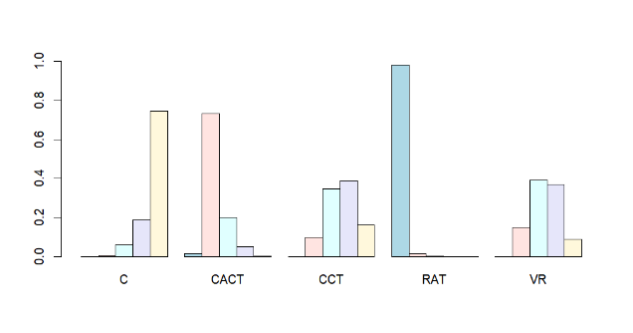
The Mini-Mental State Examination’s inclusion study stacking chart. C: conventional rehabilitation therapy; CACT: computer-assisted cognitive therapy; CCT: cognitive training; RAT: robot-assisted therapy; VR: virtual reality.

The intervention rankings are as follows: MoCA (Executive Function): CACT>VR>RAT>CCT>conventional rehabilitation therapy (C); and MMSE (Global Cognition): RAT>CACT>CCT>VR>C. See Figures 6 and 7 for a stacked diagram of the included studies.

The SUCRA score provides a relative efficacy ranking methodology that can help us identify the therapies that perform best in specific cognitive domains. High SUCRA scores indicate that a therapy may have better results in a specific cognitive domain, but this does not necessarily correspond directly to significant cognitive improvement in real-world settings, so its clinical significance should be assessed in conjunction with absolute efficacy estimates.

#### Key Comparisons

According to the league table (Tables4  5), based on the MoCA score, CACT was significantly better than C (MD=−3.02, 95% CI −4.40 to −1.35), VR was significantly superior to C (MD=−2.02, 95% CI −3.61 to −0.44), and RAT and C did not have a significant difference (MD=−1.83, 95% CI −3.99 to 0.38). Comparisons between other interventions (eg, VR vs CACT, RAT vs CCT) did not show statistical differences. Based on MMSE scores, CACT was significantly better than C (MD=−2.49, 95% CI −4.37 to −0.41), RAT was significantly better than C (MD=−6.06, 95% CI −8.94 to −2.78), RAT may be superior to CACT (MD=−3.56, 95% CI −6.69 to −0.37), RAT was significantly better than CCT (MD=−4.85, 95% CI −7.99 to −1.81), and RAT was significantly superior to VR (MD=4.86, 95% CI 1.61 to 7.69). None of the differences among VR, CCT, and C were statistically significant (CI contains 0).

### Convergence Diagnostics

After 50,000 iterations, the convergence of the network meta-analysis models was validated as follows.

On the MoCA, CACT and VR demonstrated stable trace plots and unimodal density distributions compared to conventional therapy (eg, conventional rehabilitation, occupational therapy, and drug therapy), indicating robust convergence. RAT showed minor fluctuations in trace plots and slightly wider density distributions, suggesting potential instability. However, subsequent validation confirmed model stability. The heterogeneity parameter (*I*²) exhibited a right-skewed density distribution with a peak near 0.3, indicating moderate consistency across studies.

On the MMSE, RAT achieved excellent convergence, with highly stable trace plots and sharply peaked density distributions. CCT versus conventional therapy comparisons revealed mild autocorrelation in trace plots and broader density distributions, likely due to small sample biases. Heterogeneity (*I*²) displayed a density peak near 0.5, suggesting moderate heterogeneity attributable to protocol variations or population differences (Multimedia Appendix 2).

#### Heterogeneity Analysis

Heterogeneity among the included studies was assessed in this study by the *I*² statistic. The analysis for MoCA scores showed an overall heterogeneity *I*² value of 45.22%, suggesting moderate heterogeneity between studies, while there was greater heterogeneity in MMSE scores, with an *I*² value of 76.91%. See Multimedia Appendix 3.

The potential causes of this phenomenon are discussed in depth below from a clinical perspective.

#### Heterogeneity of Intervention Programs

Dose and duration differences: the intervention duration in some studies ranged from 10 days to 3 months, and the MMSE may not be as sensitive to short-term interventions as the MoCA, which may lead to effect variability.

Differences in technology integration: in RAT LU 2022, combined gait and upper limb training, whereas XU 2023 used a speech-associated cognitive training robot, and differences in functional positioning may affect the consistency of efficacy.

#### Differences in Baseline Population Characteristics

Regarding the severity of cognitive impairment, the severity of cognitive dysfunction varied among the patients included in the study, and differences in cognitive reserve may also contribute to heterogeneity.

Regarding stroke type and site, MMSE improvement in patients with subcortical strokes (eg, basal ganglia lesions) may differ from cortical strokes, and differences in lesion site may also contribute to the variability in intervention effects.

Because of the high degree of heterogeneity, clinical interventions should be guided by careful judgments of efficacy, cost-benefit trade-offs, and the development of individualized intervention protocols that incorporate patients’ cognitive impairment characteristics.

The next step requires further analysis of sources of heterogeneity through sensitivity analysis, subgroup analysis, and subgroup regression.

### Subgroup Analysis and Meta-Regression

To understand which aspects of digital interventions may influence the effectiveness of improving cognitive function after stroke and which influences contributed to the heterogeneity of the study, a series of subgroup analyses were conducted. See Multimedia Appendix 4.

#### Patient’s Age

When the MoCA was used as an outcome indicator, the overall efficacy effect was significant in the treatment group (combined MD=1.82, 95% CI 0.96‐2.68), but the age-stratified results differed markedly.

In the age group of <65 years, efficacy was stable with minimal heterogeneity (MD=0.94, *I*²=0%), and the results had high reliability, suggesting that the results are robust in younger patients.

In the age group of ≥65 years, efficacy was stronger (MD=2.32) but heterogeneity was high (*I*²=69.2%), suggesting greater individual response variability in older patients and the need to be alert to the risk of unstable results.

When the MMSE was used as an outcome indicator, the treatment group was better than the control group overall (combined MD=1.37, 95% CI 0.43‐2.30), but the age-stratified results differed markedly.

In the age group of <65 years, efficacy was stable and heterogeneity was minimal (MD=0.94, *I*²=21.2%), and the results had high reliability, suggesting stable and generalizable efficacy in this age group.

In the age group of ≥65 years, efficacy was stronger (MD=1.11) and heterogeneity was high (73.8%, *P*<.0001), and the results were possibly unstable due to comorbidities, medication adherence, or differences in study design in older patients.

A subgroup analysis of patients of different ages revealed that the digital cognitive intervention approach was significantly age-stratified, with significant efficacy for patients younger than 65 years.

#### Intervention Characteristics

When the MoCA was used as an outcome indicator, the intervention group was better than the control group overall (combined MD=1.82, 95% CI 0.96‐2.68), but the effect differed significantly by intervention time (subgroup analysis *P*=.0104).

The analysis revealed that the efficacy of short-term interventions (6 and/or 8 weeks) was not significantly better than that of medium- and long-term interventions (3 months and/or 2 years): the effect was clear, especially in the 2-year intervention (MD=3.00, 95% CI 0.01‐5.99), which may imply that the efficacy was enhanced over time.

When the MMSE was used as an outcome indicator, the duration of the intervention may have influenced the efficacy, with clearer effects at 3 months and 10 days, but the overall results were subject to uncertainty due to high heterogeneity.

#### Meta-Regression

Meta-regression analyses showed no significant effect of intervention duration or year of publication on the impact of digital cognitive interventions to improve cognitive function outcomes after stroke (Multimedia Appendix 5).

### Sensitivity Analysis

To assess the robustness of the findings, we conducted a leave-one-out sensitivity analysis by applying R to exclude each of the included RCTs in turn. The results showed significant differences in the effect of different studies on overall heterogeneity. By excluding the study of XU 2023, we found that the heterogeneity decreased from 76.65% to 18.94%, suggesting that this study might be the main source of heterogeneity. The reason for the high heterogeneity may be that the study in XU 2023 applied language training such as the Schuell Stimulation Method, which includes overall language rehabilitation, auditory stimulation training, daily communication training, speech production training, and psychological rehabilitation. This training may not only improve the patient’s language skills, but also have a positive impact on cognitive functioning (Multimedia Appendix 6).

### Inconsistency Test and Publication Bias

Node-splitting analysis revealed no significant inconsistency between direct and indirect evidence across the network (*P*>.05 for all comparisons), confirming the stability of the consistency model. Refer to Multimedia Appendix 7.

**Figure 8.**
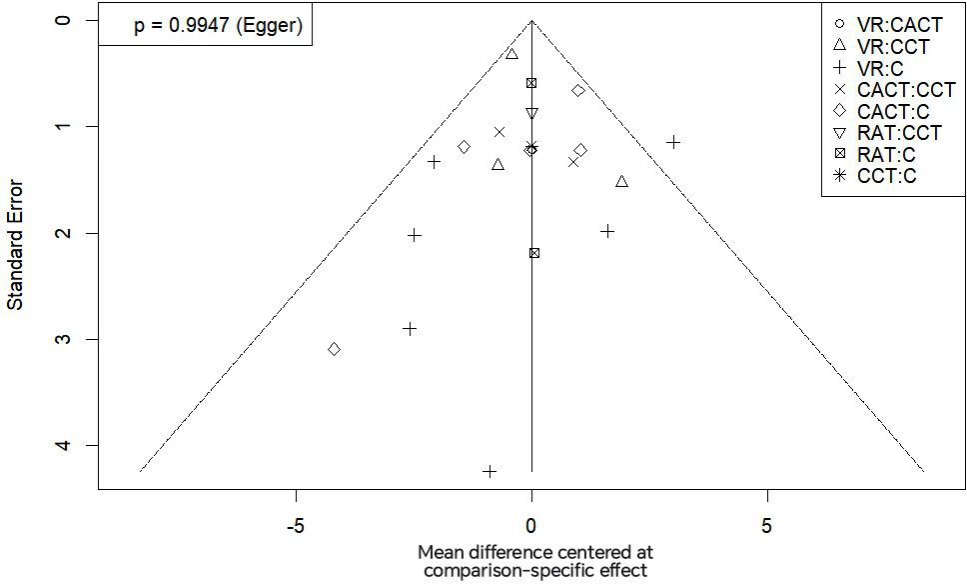
Funnel chart of the inclusion literature on the Montreal Cognitive Assessment. C: conventional rehabilitation therapy; CACT: computer-assisted cognitive therapy; CCT: cognitive training; RAT: robot-assisted therapy; VR: virtual reality.

Funnel plots for MoCA and MMSE scores exhibited symmetrical distributions, suggesting no substantial publication bias. This was further supported by Egger test results (*P*=.99 for the MoCA; *P*=.29 for the MMSE). Specific data are shown in Figures8  9.

**Figure 9.**
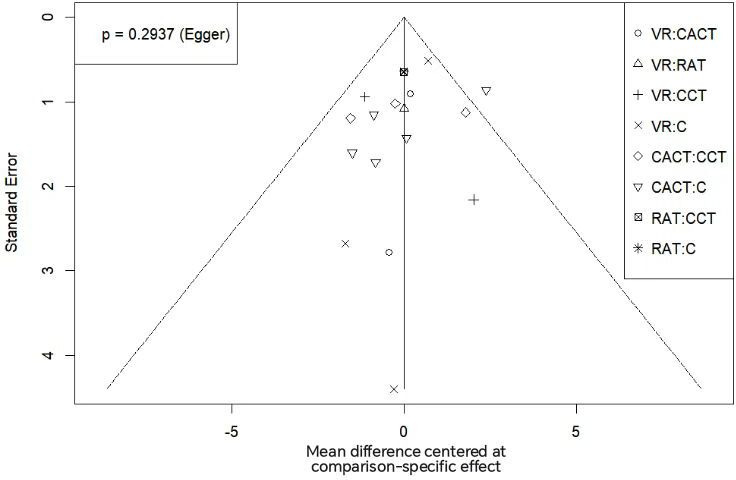
Funnel chart of the inclusion literature on the Mini-Mental State Examination. C: conventional rehabilitation therapy; CACT: computer-assisted cognitive therapy; CCT: cognitive training; RAT: robot-assisted therapy; VR: virtual reality.

### Evaluation of the Quality of Research Evidence

The quality of evidence was evaluated by applying the GRADE method, and the quality of evidence was all rated as low. The main reasons for this were the presence of biased variability, imprecision, and incoherence within the studies. Study bias was mainly due to the significant effect of high risk of bias studies on network estimates in all comparisons. Imprecision was due to insufficient data to demonstrate a conclusive effect and the fact that most comparisons were outside the 95% CI equivalence region.

## Discussion

### Summary

Poststroke cognitive impairment is a complex condition of impaired cognitive functioning that occurs after stroke [48]. It usually manifests as inattention, memory loss, slow reaction time, speech-language impairment, prosopagnosia, and so on [49], which causes great obstacles to the patient’s participation in daily life and social activities [50]. With the emergence of digital technologies such as computer-assisted therapy, VR, and tele-intervention, new directions have been brought to the assessment and treatment of cognitive impairment after stroke. Digital technologies promote neuroplasticity and functional reorganization by simulating real-life scenarios, delivering multimodal stimuli and high-intensity repetitive training [51]. In addition, digital technology can quantitatively record patient training data (eg, reaction time and error rate), providing an objective basis for dynamic assessment and program optimization [52], which is more standardized and scalable than traditional training methods.

To the best of our knowledge, this study is the first net meta-analysis that simultaneously evaluates VR, computer-assisted, and robot-assisted, and includes 27 studies involving 1517 patients, aiming to assess the impact of different digital therapies on patients with poststroke cognitive dysfunction and to rank them appropriately according to their efficacy.

### Principal Findings

The results showed that for the five digital therapies mentioned above, CACT had the highest SUCRA ranking (46/50, 91.53%) in terms of MoCA improvement, suggesting that it may be the best digital therapy for enhancing executive function, possibly because it can target activation of the prefrontal network. Xiao et al [53] showed that CACT was superior to VR in MoCA scores, with significant improvement in cognitive functions, similar to the results of this study. However, CACT therapy did not demonstrate a significant advantage in MMSE scores. The present study included more RCTs, which demonstrated that CACT has a nonnegligible effect on MMSE scores through direct and indirect evidence. The main reasons why CACT can effectively improve cognitive function in stroke patients are explained as follows: CACT relies on multimedia technology and stimulates neurons in the brain through repetitive and targeted cognitive training tasks, promoting neuroplasticity and neural network reorganization [54]. It can enhance patients’ concentration on cognitive training through dynamic visual effects and different interactive tasks, and this multisensory stimulation training can activate multiple brain regions such as prefrontal and parietal lobes [55], improving patients’ attention and executive ability. A study by Lin et al [56] found that CACT significantly improved patients’ hippocampal functional linkages with the frontal and left parietal lobes through resting-state functional magnetic resonance imaging, and that improved hippocampal and frontal lobe functioning facilitated the recovery of patients’ short-term memory and executive functioning. Bernini et al [57] found significant improvements in the overall cognitive, executive, and attentional aspects of patients who underwent CACT by comparing them with traditional paper-and-pencil training. These studies once again demonstrate the reliability of the results of the present meta-analysis. Secondly, through the application of computers, the difficulty of the task can be personalized and real-time feedback can be provided [58]. By pinpointing the areas of cognitive deficits and quantitatively recording the training data of the patients, the efficiency of the training can be effectively improved, which can help to formulate a more effective assessment and intervention plan [59].

The results showed that among the five therapies mentioned above, RAT was ranked first in SUCRA in terms of MMSE scores, indicating that robotic-assisted therapy has the best efficacy in improving MMSE scores in poststroke cognitive impairment. Robotics technology can be used to capture real-time motor imagery or visually evoked Electroencephalography signals [60], translate the patient’s active cognitive intent into action commands from the robot, and have the robot assist the patient in performing the corresponding activities. This training mode can form a closed-loop stimulation of “intention-action-feedback” [61], through which the training of closed-loop stimulation can activate key brain regions such as the primary motor cortex, hippocampus, and prefrontal lobe of the patient, and promote the patient’s memory and overall cognitive function improvement [62].

The study included 2 different outcome metrics, which were analyzed and found to yield different conclusions. On the MoCA, CACT had the best intervention effect, which may be due to the fact that the MoCA covers both visuospatial and abstract thinking dimensions and focuses more on the assessment of executive ability, whereas CACT improves executive functioning mainly through activation of the prefrontal network [63]; and thus, the MoCA is more sensitive to the therapeutic effect of CACT. The MMSE prioritizes global cognition (eg, orienting and recalling), whereas the motor-cognitive dual-task paradigm of RAT better addresses this through sensorimotor integration and hippocampal activation. Studies by Jia et al [64] and Agogo et al [65] showed that the MMSE has a “ceiling” effect on executive and recall functions, whereas the MoCA is more sensitive to the detection of executive abilities. This justifies the accuracy of the results of this study.

The effectiveness of VR for cognitive function has been controversial. Some studies [66,67] have pointed out that VR is currently focused on the improvement of motor function, and there are fewer studies on cognitive function, which makes it difficult to provide a reliable evidence-based basis. A study by Wiley et al [68] showed that the improvement of cognitive function by VR was not significantly different from that of the control group, and it only improved the patients’ initiative and sense of experience in rehabilitation. The study by Zhang et al [69] supports the viewpoint of Wiley et al [68]. This study found that VR therapy had significant efficacy on the MoCA but little efficacy in the MMSE.

The differential efficacy of VR across the MoCA and MMSE outcomes likely arises from both mechanistic and methodological factors. Mechanistically, the MoCA emphasizes executive function (eg, working memory, cognitive flexibility, and visuospatial reasoning), which aligns with VR’s capacity to deliver task-specific challenges requiring real-time decision-making and adaptive problem-solving [67]. For example, VR systems like Reh@City integrate dual-task paradigms (eg, navigating virtual environments while solving arithmetic problems), which selectively activate the dorsolateral prefrontal cortex and posterior parietal regions—key neural substrates for executive function [70]. These targeted activations may explain VR’s superior performance in MoCA scores. Conversely, the MMSE prioritizes global cognitive domains such as orientation, memory recall, and language comprehension—functions less directly engaged by current VR protocols. Most VR interventions in this analysis focused on motor-cognitive integration (eg, reaching tasks in virtual environments), which may inadequately stimulate hippocampal and medial temporal networks critical for MMSE-assessed domains.

Methodologically, heterogeneity in VR protocols further complicates interpretation. Studies demonstrating VR benefits for the MoCA; for example, Faria et al [24] and Rogers et al [26] used adaptive difficulty systems and multisensory feedback, whereas trials with null MMSE outcomes, for instance, those of Choi et al [40] and Oh et al [28] used simpler VR tasks lacking cognitive progression or sensory feedback. In addition, the MMSE’s ceiling effects in mild cognitive impairment populations [71] and its insensitivity to executive improvements may underestimate VR’s impact on higher-order cognition. Second, cognition consists of several aspects, such as memory, executive ability, etc [72]. Only by corresponding to specific areas can a certain cognitive function be effectively improved. Perhaps a VR system that truly focuses on cognitive rehabilitation can change the controversial situation of VR for improving cognition.

There are significant differences in the cognitive domains assessed by the MoCA and the MMSE. The MoCA focuses on assessing higher cognitive functions such as executive functioning, attention, and visuospatial abilities, while the MMSE focuses primarily on basic cognitive functions such as orientation, memory, and language. Therefore, they have different sensitivities and specificities in assessing cognitive impairment.

In the study, it was found that CACT showed a significant effect in improving executive functioning through activation of the prefrontal network, which was reflected in the MoCA scores. However, this effect may not directly translate into significant improvements in MMSE scores, which are more focused on basic cognitive functions. Similarly, the RAT activates the motor cortex and hippocampus through a dual motor-cognitive task paradigm, thereby showing benefits in global cognitive function as assessed by the MMSE, but may not be as effective as the CACT in executive function as assessed by the MoCA. So different interventions may be effective in specific cognitive domains, but not necessarily for all cognitive impairments.

The study’s findings provide actionable insights for integrating digital therapies into clinical practice, contingent on balancing efficacy, accessibility, and patient-specific factors.

CACT is optimal for patients with executive dysfunction (prioritize MoCA-based deficits). Most CACT applications have software license fees (eg, the Reh@Com system), but have low hardware requirements and are reusable for patients with long-term interventions. Their personalized algorithms can reduce labor inputs and are cost-effective and scalable in resource-limited areas.

RAT has a high initial acquisition cost, but it can integrate motor and cognitive training and reduce multiple equipment investments. This meta-analysis showed that RAT had the highest SUCRA score (99.44%) for the MMSE, suggesting that it is efficient in improving patients’ overall cognitive function and may have long-term cost-effectiveness. Despite the high initial investment, RAT plays a critical role in long-term rehabilitation and may offset the initial cost. In the clinic, this integrated training approach increases treatment efficiency, reduces the cost of switching patients between multiple devices, and improves the rehabilitation experience and adherence. Thus, in the long term, RAT excels in cognitive rehabilitation and may be a cost-effective option.

Although VR technology has gained attention for its highly engaging and superior visuospatial training effects, it faces some challenges in clinical applications. For example, the high cost of immersive VR devices (eg, Oculus Quest 2) and associated scenario development may limit its widespread use in certain resource-limited health care organizations.

However, it is worth pointing out that the efficiency of immersive training can somewhat compensate for its costly investment. By providing a highly immersive training environment, VR technology can significantly reduce the time required for a single training session while improving the effectiveness of the training. This efficiency not only helps patients recover cognitive functions faster, but also indirectly reduces the total cost of rehabilitation and care in the long run. Thus, despite the problem of high initial costs, the benefits of this technology may far outweigh its costs in the long run, especially in cases where frequent training and long-term interventions are required.

Patient preferences also play an important role in the application of therapy. For example, younger patients or those who are more receptive to technology may prefer more interactive digital therapies such as VR or CACT, whereas older patients or those who are unfamiliar with technology may be more comfortable with traditional cognitive training or RAT. In addition, the type and severity of a patient’s cognitive impairment may also affect their acceptance of and adherence to different therapies. Therefore, digital cognitive intervention methods should consider the above factors in the process of clinical application and develop individualized programs suitable for patients.

### Limitations

This study provides valuable insights into the efficacy of digital therapies for PSCI, but several limitations must be acknowledged. First, the GRADE framework rated the quality of evidence as low across all comparisons, primarily due to three interrelated issues: (1) risk of bias: approximately 26% of included studies exhibited high risk of bias, particularly in blinding of participants/personnel and allocation concealment. For example, unblinded therapists may have inadvertently influenced patient engagement in VR or RAT interventions, potentially inflating effect estimates for these therapies compared to conventional rehabilitation. (2) Imprecision: wide CIs in key comparisons reflect insufficient statistical power to distinguish small but clinically meaningful differences, likely due to small sample sizes in robotic trials (n=4 studies). (3) Incoherence: Heterogeneity persisted despite sensitivity analyses, attributable to variations in intervention protocols (eg, adaptive vs fixed-difficulty VR systems) and population characteristics (eg, inclusion of both subacute and chronic stroke phases).

These limitations have important implications for clinical implementation. Although CACT and RAT demonstrated domain-specific superiority in MoCA and MMSE scores, respectively, the lower GRADE scores suggest that clinicians should view the study’s findings with caution. For example, the apparent superiority of the RAT on the MMSE may be influenced by its frequent combination with motor training (eg, the Lokomat system), which may synergistically enhance overall cognitive performance through activation of sensory-motor networks, a mechanism that has not been isolated in the current RAT trial.

In addition, this study mainly focused on the short-term treatment effect and lacked long-term follow-up data to comprehensively assess the long-term efficacy of digital therapies and the long-term prognosis of patients, precluding conclusions regarding sustained cognitive benefits or cost-effectiveness.

### Future Directions

Given these limitations, future research should focus on the following areas. First, it is crucial to conduct long-term follow-up studies. This will help to gain insights into the long-term effects of digital therapies on patients with PSCI and assess their lasting effects in delaying cognitive decline, improving quality of life, and promoting social regression. Second, future studies should further optimize the intervention protocols, including clarifying the optimal intervention dose, frequency, and duration of different digital therapies, as well as exploring the basis for the development of individualized treatment plans. In addition, combining neuromodulation techniques or traditional rehabilitation means, such as transcranial magnetic stimulation and acupuncture, with digital therapies for synergistic application may bring more significant rehabilitation benefits to patients, which is worth exploring in depth. Finally, from the perspective of technological development, with the continuous advancement of AI, VR, and other technologies, how to ensure that digital therapies reduce costs and maintain therapeutic efficacy through continuous technological updating and optimization is also an important area of research. For example, in resource-limited settings, CACT delivered via tablet devices can provide a cost-effective cognitive rehabilitation program. And reinforcement learning algorithms can be integrated into VR-based cognitive training modules to dynamically adjust task difficulty based on real-time performance. Future research should develop smarter adaptive training systems, more immersive VR scenarios, and more accurate tools for monitoring and analyzing patient data in order to further improve the effectiveness and feasibility of digital therapies in the field of poststroke cognitive rehabilitation.

### Conclusion

Overall, digital therapies provide scientific and personalized solutions for poststroke cognitive rehabilitation, with CACT and RAT demonstrating domain-specific advantages in executive function and global cognition, respectively. These findings align with the study’s aims to quantify relative efficacy and identify optimal strategies for specific cognitive domains. However, heterogeneity and methodological limitations in existing trials necessitate further validation through high-quality RCTs with standardized protocols, larger samples, and long-term follow-ups. Clinical translation requires balancing efficacy, safety, and cost-effectiveness to advance precision rehabilitation strategies.

## Supplementary material

10.2196/73687 Multimedia Appendix 1 Search strategy.

10.2196/73687 Multimedia Appendix 2 Convergence diagnostics.

10.2196/73687 Multimedia Appendix 3 Heterogeneity analysis.

10.2196/73687 Multimedia Appendix 4 Subgroup analysis.

10.2196/73687 Multimedia Appendix 5 Meta-regression.

10.2196/73687 Multimedia Appendix 6 Sensitivity analysis.

10.2196/73687 Multimedia Appendix 7 Node-splitting analysis.

10.2196/73687 Checklist 1 PRISMA (Preferred Reporting Items for Systematic Reviews and Meta-Analyses) checklist.

## Abbreviations

AI: artificial intelligence
C: conventional rehabilitation therapy
CACT: computer-assisted cognitive therapy
CCT: cognitive training
DIC: Deviance Information Criterion
GRADE: Grading of Recommendations Assessment, Development, and Evaluation
MD: mean difference
MeSH: Medical Subject Headings
MMSE: Mini-Mental State Examination
MoCA: Montreal Cognitive Assessment
NMA: network meta-analysis
PICOS: Population, Intervention, Control, Outcome, and Study Design
PRISMA: Preferred Reporting Items for Systematic Reviews and Meta-Analyses
PSCI: poststroke cognitive impairment
RAT: robot-assisted therapy
RCT: randomized controlled trial
SUCRA: Surface Under the Cumulative Ranking Curve
VR: virtual reality
