# Supplementary material for: Digital Interventions for Cognitive Dysfunction in Patients With Stroke: Systematic Review and Meta-Analysis
Source: J Med Internet Res. 2025 Jul 24;27:e73687. doi: 10.2196/73687 (PMC12288705; doi:10.2196/73687)
Supplement: Multimedia Appendix 1 [file jmir-v27-e73687-s001.docx]

**Multimedia Appendix 1.**Search Strategy

**Pubmed**

| **#** | **Search terms** | **Results** |
| --- | --- | --- |
| 1 | Stroke[MeSH Terms] | 189,272 |
| 2 | stroke[Title/Abstract] OR Strokes[Title/Abstract] OR Cerebrovascular Accident[Title/Abstract] OR Cerebrovascular Accidents[Title/Abstract] OR Cerebral Stroke[Title/Abstract] OR Cerebral Strokes[Title/Abstract] OR Stroke, Cerebral[Title/Abstract] OR Strokes, Cerebral[Title/Abstract] OR Cerebrovascular Apoplexy[Title/Abstract] OR Apoplexy, Cerebrovascular[Title/Abstract] OR Vascular Accident, Brain[Title/Abstract] OR Brain Vascular Accident[Title/Abstract] OR Brain Vascular Accidents[Title/Abstract] OR Vascular Accidents, Brain[Title/Abstract] OR Cerebrovascular Stroke[Title/Abstract] OR Cerebrovascular Strokes[Title/Abstract] OR Stroke, Cerebrovascular[Title/Abstract] OR Strokes, Cerebrovascular[Title/Abstract] OR Apoplexy[Title/Abstract] OR Acute Strokes[Title/Abstract] OR Strokes, Acute[Title/Abstract] OR Cerebrovascular Accident, Acute[Title/Abstract] OR Acute Cerebrovascular Accident[Title/Abstract] OR Acute Cerebrovascular Accidents[Title/Abstract] | 366,172 |
| 3 | Cognition[MeSH Terms] | 215,765 |
| 4 | Cognition[Title/Abstract] OR Disorder, Cognition[Title/Abstract] OR Disorders, Cognition[Title/Abstract] OR Overinclusion[Title/Abstract] | 127,883 |
| 5 | Artificial Intelligence[MeSH Terms] | 227,122 |
| 6 | Intelligence, Artificial[Title/Abstract] OR Computer Reasoning[Title/Abstract] OR Reasoning, Computer[Title/Abstract] OR AI (Artificial Intelligence[Title/Abstract]) OR Machine Intelligence[Title/Abstract] OR Intelligence, Machine[Title/Abstract] OR Computational Intelligence[Title/Abstract] OR Intelligence, Computational[Title/Abstract] OR Computer Vision Systems[Title/Abstract] OR Computer Vision System[Title/Abstract] OR System, Computer Vision[Title/Abstract] OR Systems, Computer Vision[Title/Abstract] OR Intelligence, Artificial[Title/Abstract] OR Computer Reasoning[Title/Abstract] OR Reasoning, Computer[Title/Abstract] OR AI (Artificial Intelligence[Title/Abstract]) OR Machine Intelligence[Title/Abstract] OR Intelligence, Machine[Title/Abstract] OR Computational Intelligence[Title/Abstract] OR Intelligence, Computational[Title/Abstract] OR Computer Vision Systems[Title/Abstract] OR Computer Vision System[Title/Abstract] OR System, Computer Vision[Title/Abstract] OR Systems, Computer Vision[Title/Abstract] | 31,978 |
| 7 | Virtual Reality[MeSH Terms] | 8,148 |
| 8 | Reality, Virtual[Title/Abstract] OR Virtual Reality, Educational[Title/Abstract] OR Educational Virtual Realities[Title/Abstract] OR Educational Virtual Reality[Title/Abstract] OR Reality, Educational Virtual[Title/Abstract] OR Virtual Realities, Educational[Title/Abstract] OR Virtual Reality, Instructional[Title/Abstract] OR Instructional Virtual Realities[Title/Abstract] OR Instructional Virtual Reality[Title/Abstract] OR Realities, Instructional Virtual[Title/Abstract] OR Reality, Instructional Virtual[Title/Abstract] OR Virtual Realities, Instructional[Title/Abstract] OR Virtual Reality[Title/Abstract] | 22,571 |
| 9 | Therapy, Computer-Assisted[MeSH Terms] | 48,399 |
| 10 | Therapy, Computer Assisted[Title/Abstract] OR Computer-Assisted Protocol-Directed Therapy[Title/Abstract] OR Computer-Assisted Protocol-Directed Therapies[Title/Abstract] OR Computer Assisted Protocol Directed Therapy[Title/Abstract] OR Protocol-Directed Therapies, Computer-Assisted[Title/Abstract] OR Therapies, Computer-Assisted Protocol-Directed[Title/Abstract] OR Therapy, Computer-Assisted Protocol-Directed[Title/Abstract] OR Therapy, Computer Assisted Protocol Directed[Title/Abstract] OR Protocol-Directed Therapy, Computer-Assisted[Title/Abstract] OR Protocol Directed Therapy, Computer Assisted[Title/Abstract] OR Computer-Assisted Therapy[Title/Abstract] OR Computer-Assisted Therapies[Title/Abstract] OR Computer Assisted Therapy[Title/Abstract] OR Therapies, Computer-Assisted[Title/Abstract] | 11,310 |
| 11 | Robotics[MeSH Terms] | 47,717 |
| 12 | Remote Operations (Robotics[Title/Abstract]) OR Operation, Remote (Robotics[Title/Abstract]) OR Remote Operation (Robotics[Title/Abstract]) OR Telerobotics[Title/Abstract] OR Telerobotics[Title/Abstract] OR robot[Title/Abstract] OR Humanoid Robots[Title/Abstract] | 37,808 |
| 13 | #1 OR #2 | 405,096 |
| 14 | #3 OR #4 | 301,800 |
| 15 | #5 OR #6 | 242,189 |
| 16 | #7 OR #8 | 23,921 |
| 17 | #9 OR #10 | 58,860 |
| 18 | #11 OR #12 | 65,141 |
| 19 | #13 AND #14 | 7,954 |
| 20 | #15 OR #16 OR #17 OR #18 | 353,738 |
| 21 | #19 AND #20 | 226 |

**Cochrane Library**

| **#** | **Search terms** | **Results** |
| --- | --- | --- |
| 1 | MeSH descriptor: [Stroke] explode all trees | 17,698 |
| 2 | Stroke OR strokeS OR Cerebrovascular Accident OR Cerebrovascular Accidents OR Cerebral Stroke OR Cerebral Strokes OR Stroke, Cerebral OR Strokes, Cerebral OR Cerebrovascular Apoplexy OR Apoplexy, Cerebrovascular OR Vascular Accident, Brain OR Brain Vascular Accident OR Brain Vascular Accidents OR Vascular Accidents, Brain OR Cerebrovascular Stroke OR Cerebrovascular Strokes OR Stroke, Cerebrovascular OR Strokes, Cerebrovascular OR Apoplexy OR Acute Strokes OR Strokes, Acute OR Cerebrovascular Accident, Acute OR Acute Cerebrovascular Accident OR Acute Cerebrovascular Accidents | 93,525 |
| 3 | #1 OR #2 | 93,903 |
| 4 | MeSH descriptor: [Cognition] explode all trees | 16,253 |
| 5 | Cognition Disorders OR Disorder, Cognition OR Disorders, Cognition OR Overinclusion OR Cognition | 40,117 |
| 6 | #4 OR #5 | 46,137 |
| 7 | MeSH descriptor: [Artificial Intelligence] explode all trees | 3,388 |
| 8 | Intelligence, Artificial OR Computer Reasoning OR Reasoning, Computer OR AI (Artificial Intelligence) OR Machine Intelligence OR Intelligence, Machine OR Computational Intelligence OR Intelligence, Computational OR Computer Vision Systems OR Computer Vision System OR System, Computer Vision OR Systems, Computer Vision OR Intelligence, Artificial OR Computer Reasoning OR Reasoning, Computer OR AI (Artificial Intelligence) OR Machine Intelligence OR Intelligence, Machine OR Computational Intelligence OR Intelligence, Computational OR Computer Vision Systems OR Computer Vision System OR System, Computer Vision OR Systems, Computer Vision | 4,068 |
| 9 | #7 OR #8 | 6,622 |
| 10 | MeSH descriptor: [Virtual Reality] explode all trees | 1,304 |
| 11 | Reality, Virtual OR Virtual Reality, Educational OR Educational Virtual Realities OR Educational Virtual Reality OR Reality, Educational Virtual OR Virtual Realities, Educational OR Virtual Reality, Instructional OR Instructional Virtual Realities OR Instructional Virtual Reality OR Realities, Instructional Virtual OR Reality, Instructional Virtual OR Virtual Realities, Instructional OR Virtual Reality | 8,237 |
| 12 | #10 OR #11 | 8,250 |
| 13 | MeSH descriptor: [Therapy, Computer-Assisted] explode all trees | 3,145 |
| 14 | Therapy, Computer Assisted OR Computer-Assisted Protocol-Directed Therapy OR Computer-Assisted Protocol-Directed Therapies OR Computer Assisted Protocol Directed Therapy OR Protocol-Directed Therapies, Computer-Assisted OR Therapies, Computer-Assisted Protocol-Directed OR Therapy, Computer-Assisted Protocol-Directed OR Therapy, Computer Assisted Protocol Directed OR Protocol-Directed Therapy, Computer-Assisted OR Protocol Directed Therapy, Computer Assisted OR Computer-Assisted Therapy OR Computer-Assisted Therapies OR Computer Assisted Therapy OR Therapies, Computer-Assisted | 25,877 |
| 15 | #13 OR #14 | 26,618 |
| 16 | MeSH descriptor: [Robotics] explode all trees | 2,042 |
| 17 | Remote Operations (Robotics) OR Operation, Remote (Robotics) OR Remote Operation (Robotics) OR Telerobotics OR Telerobotics OR robot OR Humanoid Robots | 3,052 |
| 18 | #16 OR #17 | 3,785 |
| 19 | #3 AND #6 | 3,032 |
| 20 | #9 OR #12 OR #15 OR #18 | 42,069 |
| 21 | #19 AND #20 | 374 |

**Web of Science**

| **#** | **Search terms** | **Results** |
| --- | --- | --- |
| 1 | TS=(Strokes OR Cerebrovascular Accident OR Cerebrovascular Accidents OR Cerebral Stroke OR Cerebral Strokes OR Stroke, Cerebral OR Strokes, Cerebral OR Cerebrovascular Apoplexy OR Apoplexy, Cerebrovascular OR Vascular Accident, Brain OR Brain Vascular Accident OR Brain Vascular Accidents OR Vascular Accidents, Brain OR Cerebrovascular Stroke OR Cerebrovascular Strokes OR Stroke, Cerebrovascular OR Stroke) | 421,139 |
| 2 | TS=(Disorder, Cognition OR Disorders, Cognition OR Overinclusion OR Cognition Disorders OR Cognition) | 184,764 |
| 3 | TS=(Reality, Virtual OR Virtual Reality, Educational OR Educational Virtual Realities OR Educational Virtual Reality OR Reality, Educational Virtual OR Virtual Realities, Educational OR Virtual Reality, Instructional OR Instructional Virtual Realities OR Instructional Virtual Reality OR Realities, Instructional Virtual OR Reality, Instructional Virtual OR Virtual Realities, Instructional OR Virtual Reality) | 44,586 |
| 4 | TS=(Intelligence, Artificial OR Computer Reasoning OR Reasoning, Computer OR AI (Artificial Intelligence) OR Machine Intelligence OR Intelligence, Machine OR Computational Intelligence OR Intelligence, Computational OR Computer Vision Systems OR Computer Vision System OR System, Computer Vision OR Systems, Computer Vision OR Intelligence, Artificial OR Computer Reasoning OR Reasoning, Computer OR AI (Artificial Intelligence) OR Machine Intelligence OR Intelligence, Machine OR Computational Intelligence OR Intelligence, Computational OR Computer Vision Systems OR Computer Vision System OR System, Computer Vision OR Systems, Computer Vision) | 175,516 |
| 5 | TS=(Therapy, Computer Assisted OR Computer-Assisted Protocol-Directed Therapy OR Computer-Assisted Protocol-Directed Therapies OR Therapies, Computer-Assisted OR cognitive training) | 48,231 |
| 6 | TS=(ROBOT OR Robotics OR Remote Operations ) | 230,120 |
| 7 | #1 AND #2 | 5347 |
| 8 | #3 OR #4 OR #5 OR #6 | 480,552 |
| 9 | #7 AND #8 | 411 |

**Scopus(N=161)**

## ( TITLE-ABS-KEY ( robots  OR  virtual  AND reality  OR  computer-assisted )  AND  TITLE-ABS-KEY ( stroke  OR  strokes  OR  cerebrovascular  AND  accident  OR  cerebrovascular  AND  accidents  OR  cerebral  AND  stroke  OR  cerebral  AND  strokes )  AND  TITLE-ABS-KEY ( cognition  AND disorders  OR  cognition ) )

EMbase

| **#** | **Search terms** | **Results** |
| --- | --- | --- |
| 1 | 'cognitive defect'/exp OR 'cognitive defect' | 699,770 |
| 2 | ((disorder, AND cognition OR disorders,) AND cognition OR overinclusion OR cognition) AND disorders | 83,193 |
| 3 | #1 OR #2 | 753,412 |
| 4 | 'cerebrovascular accident'/exp OR 'cerebrovascular accident' | 496,924 |
| 5 | 'cerebrovascular accident'/exp OR 'cerebrovascular accident' | 8267 |
| 6 | #4 OR #5 | 499,365 |
| 7 | 'virtual reality'/exp OR 'virtual reality' | 41,854 |
| 8 | ((((((((((reality, AND virtual OR virtual) AND reality, AND educational OR educational) AND virtual AND realities OR educational virtual) AND reality OR reality,) AND educational AND virtual OR virtual) AND realities, AND educational OR virtual) AND reality, AND instructional OR instructional) AND virtual AND realities OR instructional virtual) AND reality OR realities,) AND instructional AND virtual OR reality,) AND instructional AND virtual OR virtual | 526,362 |
| 9 | #7 OR #8 | 526,362 |
| 10 | 'computer assisted therapy'/exp OR 'computer assisted therapy' | 15,433 |
| 11 | (((((((((((((therapy, AND computer AND assisted OR 'computer assisted') AND 'protocol directed' AND therapy OR 'computer assisted') AND 'protocol directed' AND therapies OR computer) AND assisted AND protocol AND directed AND therapy OR 'protocol directed') AND therapies, AND 'computer assisted' OR therapies,) AND 'computer assisted' AND 'protocol directed' OR therapy,) AND 'computer assisted protocol directed' OR therapy,) AND computer AND assisted protocol AND directed OR 'protocol directed therapy,') AND 'computer assisted' OR protocol) AND directed therapy, AND computer AND assisted OR 'computer assisted') AND therapy OR 'computer assisted') AND therapies OR computer) AND assisted AND therapy OR therapies,) AND 'computer assisted' | 447,464 |
| 12 | #10 OR #11 | 450,179 |
| 13 | 'robotics'/exp OR 'robotics' | 74,498 |
| 14 | robotics OR robot | 123,859 |
| 15 | #13 OR #14 | 123,998 |
| 16 | #3 AND #6 | 370,23 |
| 17 | #9 OR #12 OR #15 | 1,086,982 |
| 18 | #16 AND #17 | 1614 |
| 19 | AND ('controlled study'/de OR 'randomized controlled trial'/de) | 580 |

**CNKI(N=259)**

**((SU=脑卒中 + 脑卒中患者 + 脑卒中康复 + 缺血性脑卒中 + 缺血性脑卒中患者) AND (SU=认知 + 认知功能 + 认知能力 + 认知功能障碍 + 认知障碍)) AND ((SU=虚拟现实 + 虚拟现实技术 + 虚拟现实系统 + '虚拟现实(vr)' + 虚拟现实技术应用 + '虚拟现实(vr)技术') OR (SU=计算机辅助 + 计算机辅助技术) OR (SU=机器人 + 机器人系统 + 机器人技术 + 机器人辅助))**
