# Supplementary figures and images for: Digital Interventions for Cognitive Dysfunction in Patients With Stroke: Systematic Review and Meta-Analysis
Source: J Med Internet Res. 2025 Jul 24;27:e73687. doi: 10.2196/73687 (PMC12288705; doi:10.2196/73687)

**Convergence Diagnostic Chart(*MOCA*)**


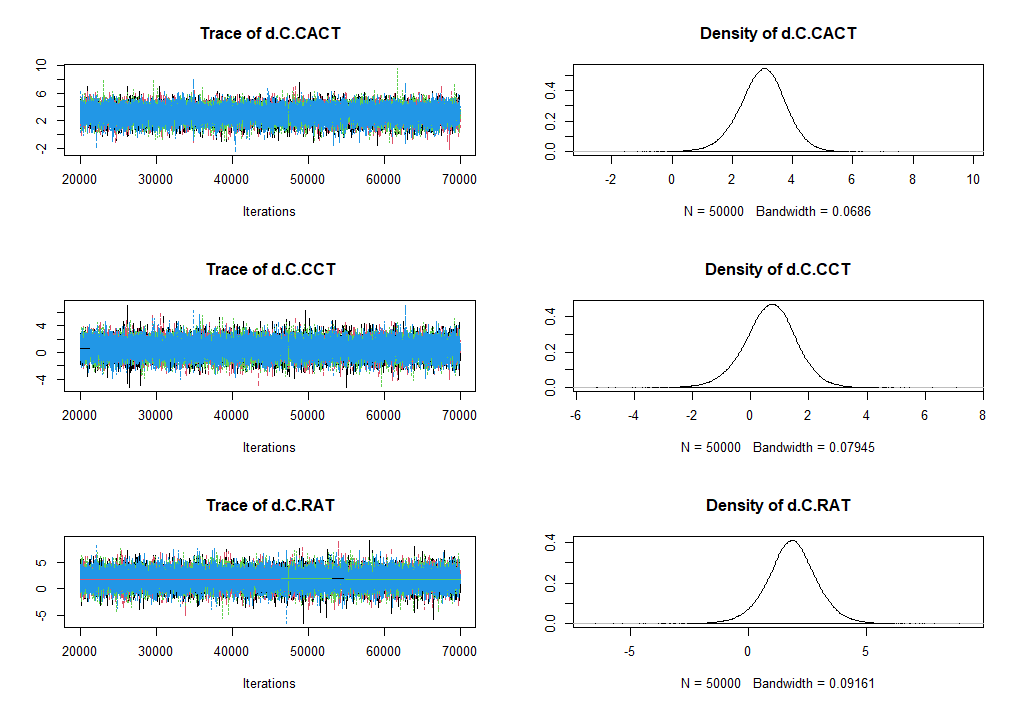

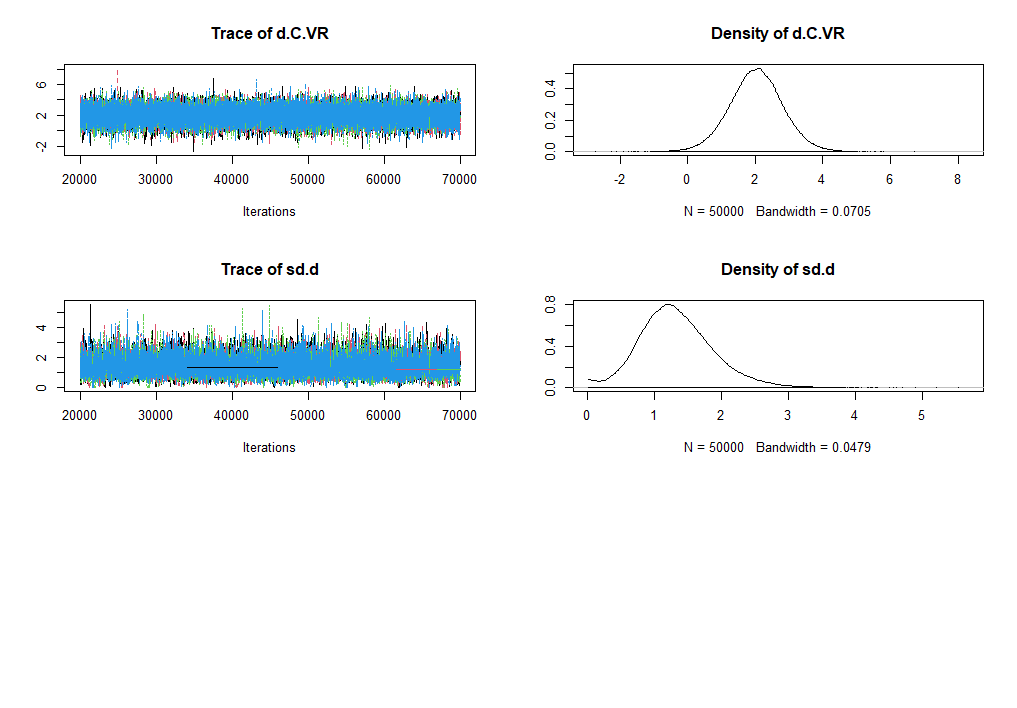

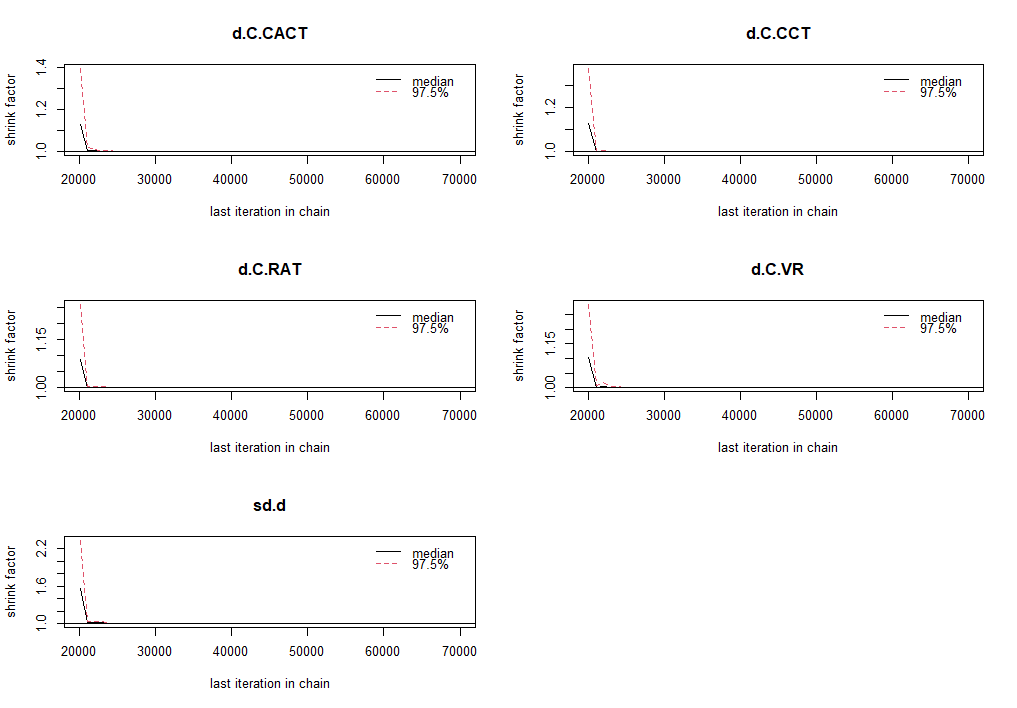


**Convergence Diagnostic Chart(*MMSE*)**


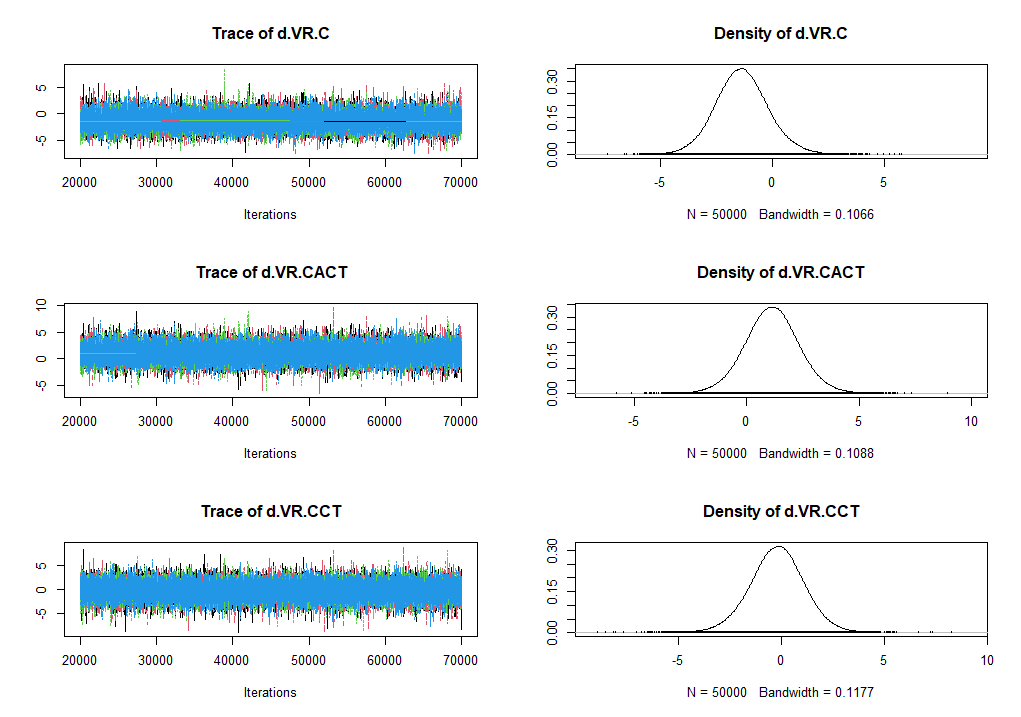

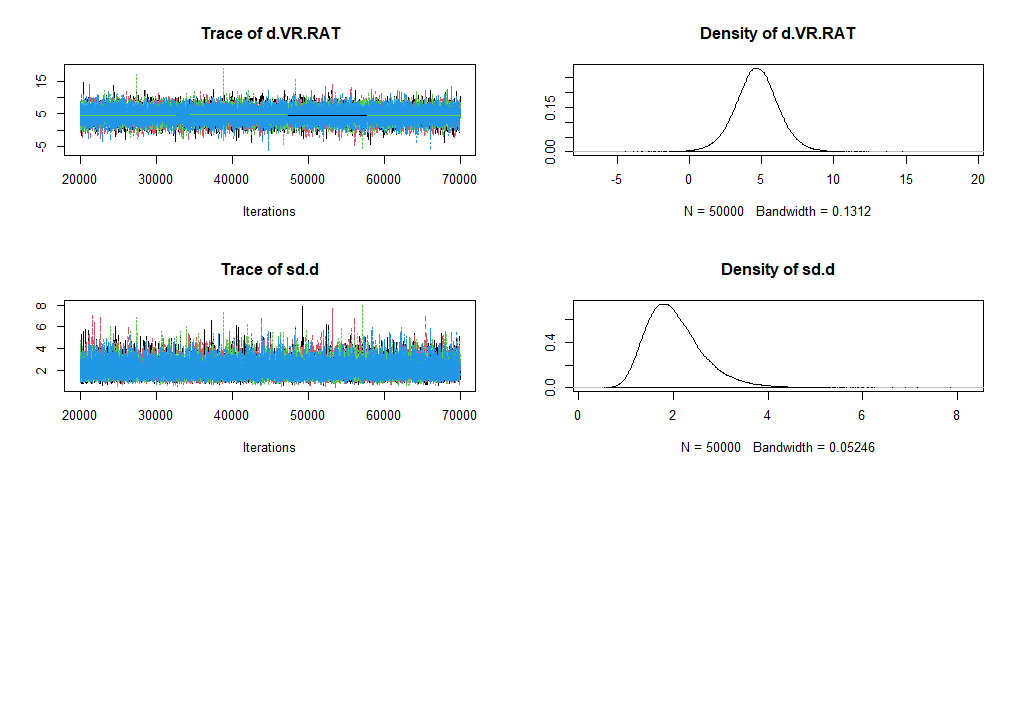

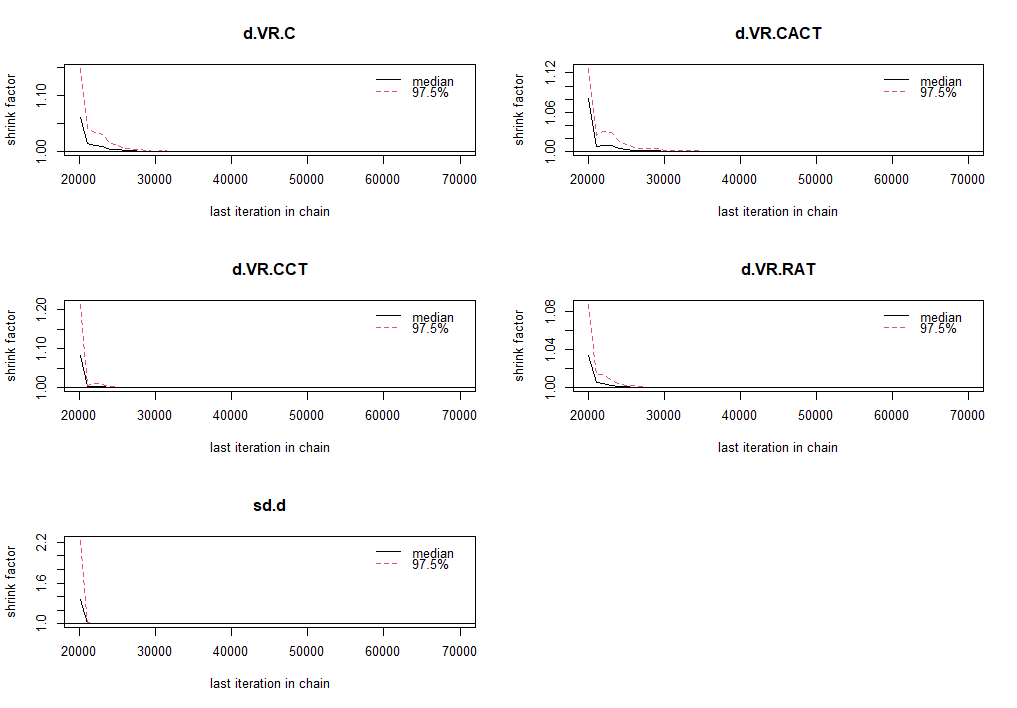

Supplement: Multimedia Appendix 2 [file jmir-v27-e73687-s002.docx]

Heterogeneity Analysis(MOCA)


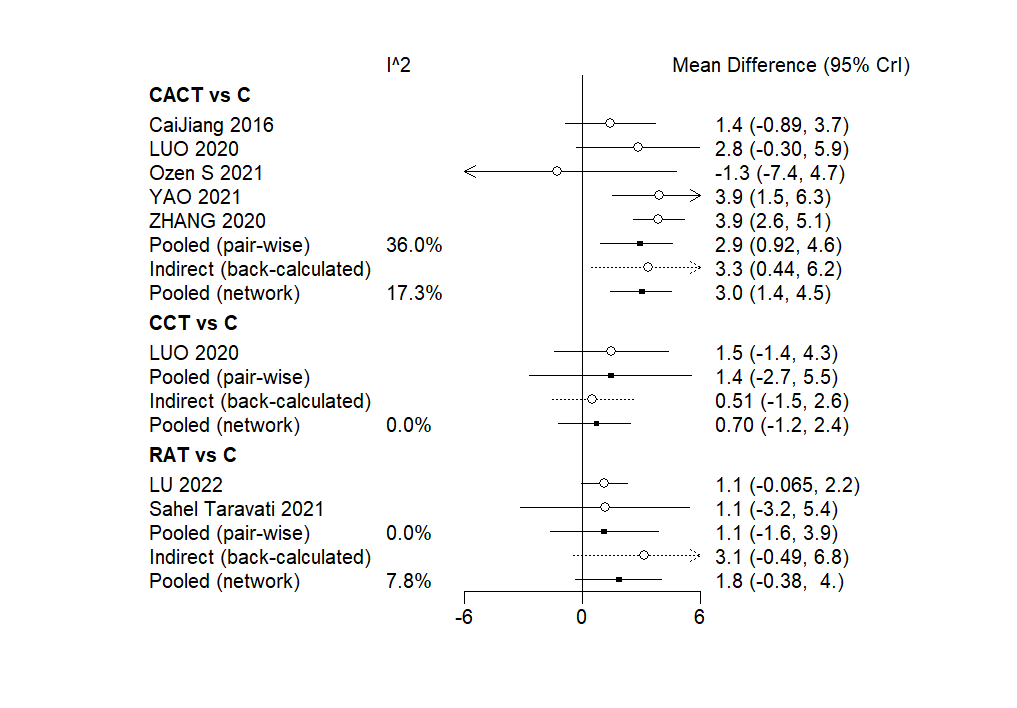

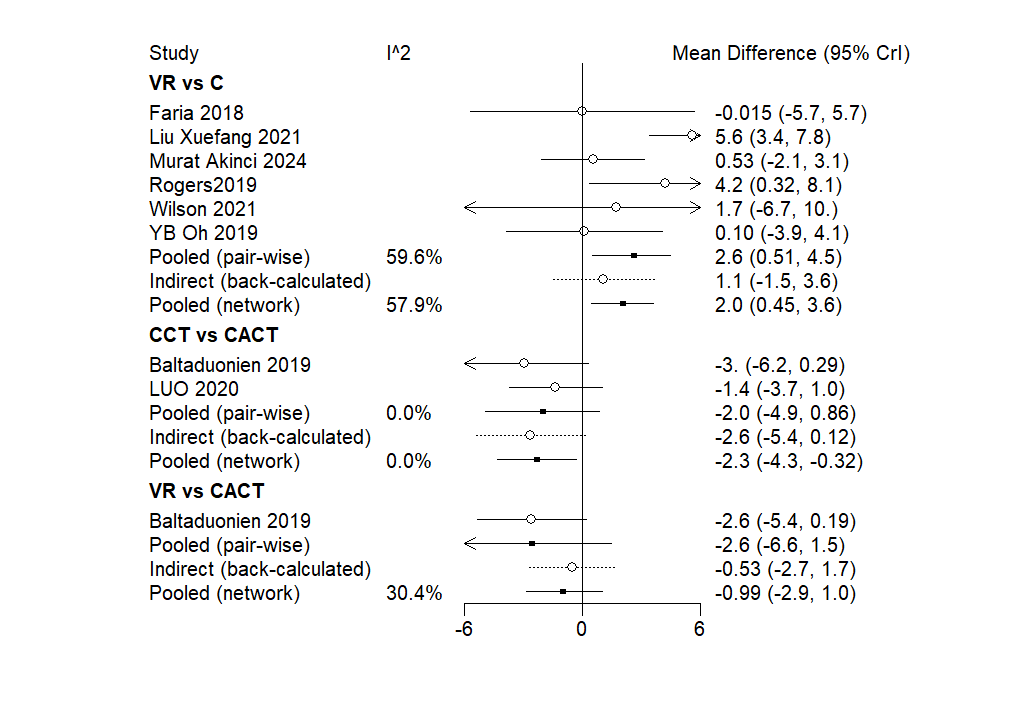

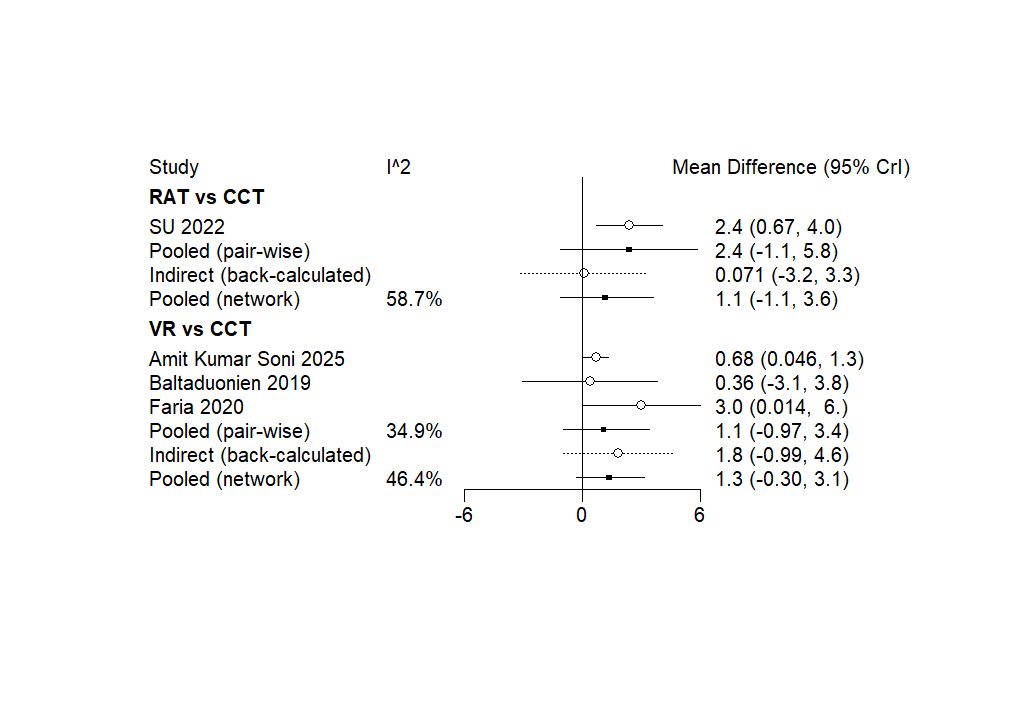


Heterogeneity Analysis(MMSE)


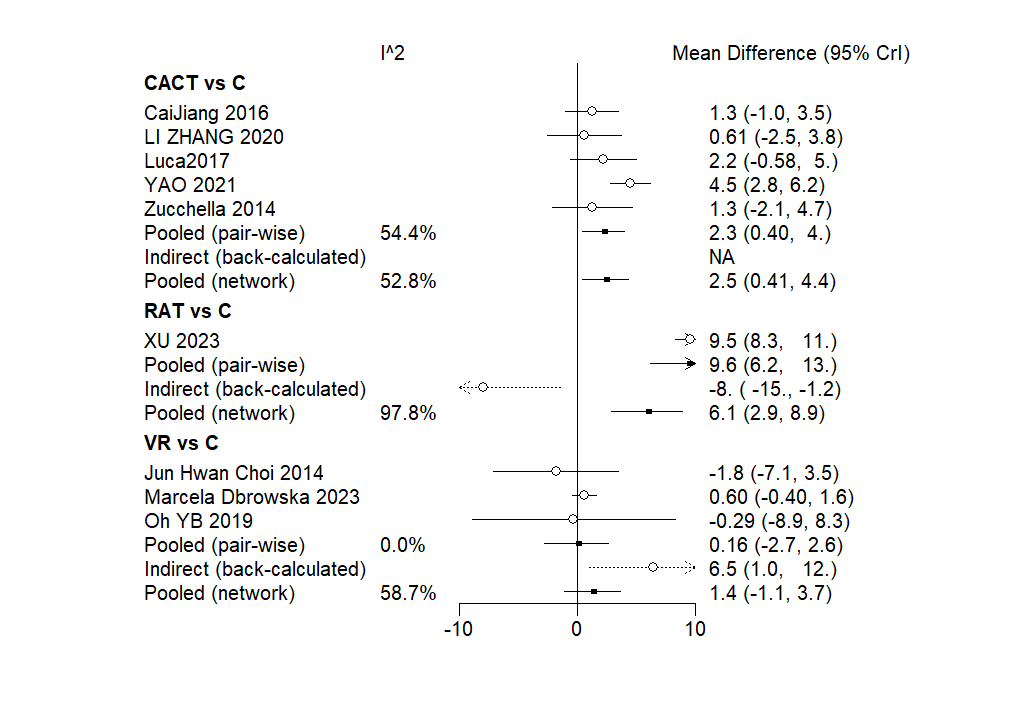

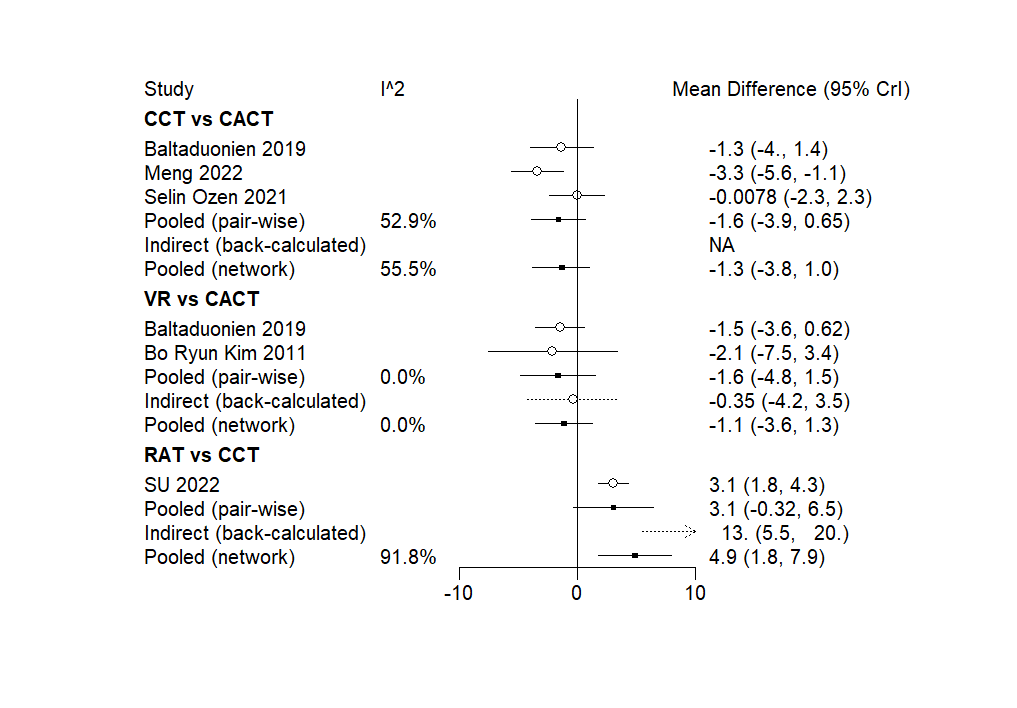

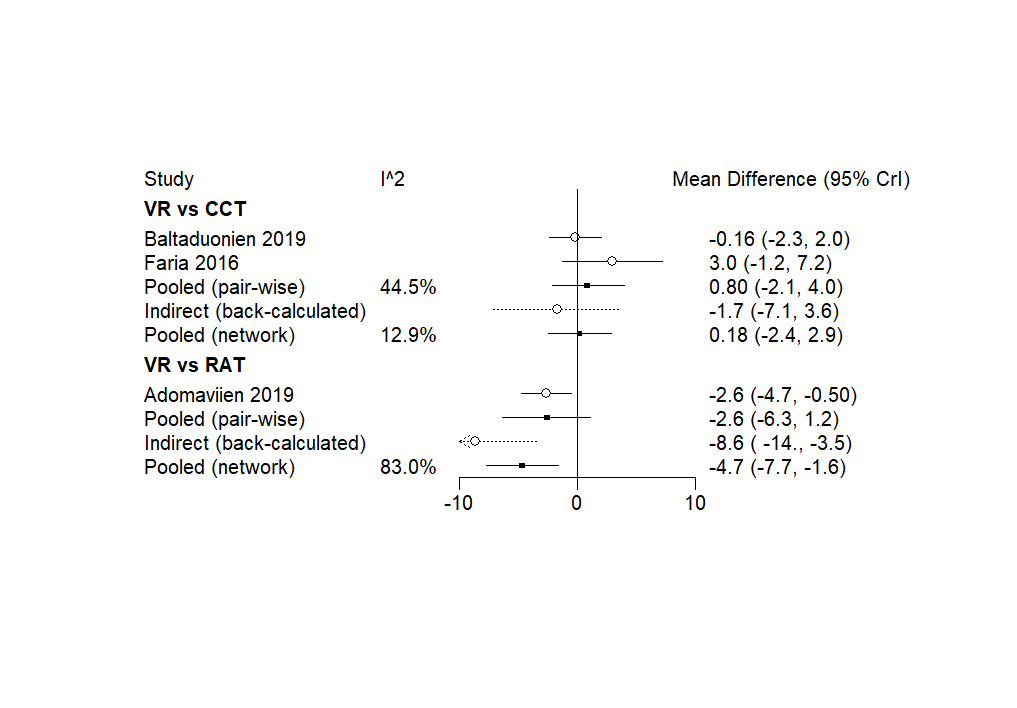

Supplement: Multimedia Appendix 3 [file jmir-v27-e73687-s003.docx]

Meta-regression(year)


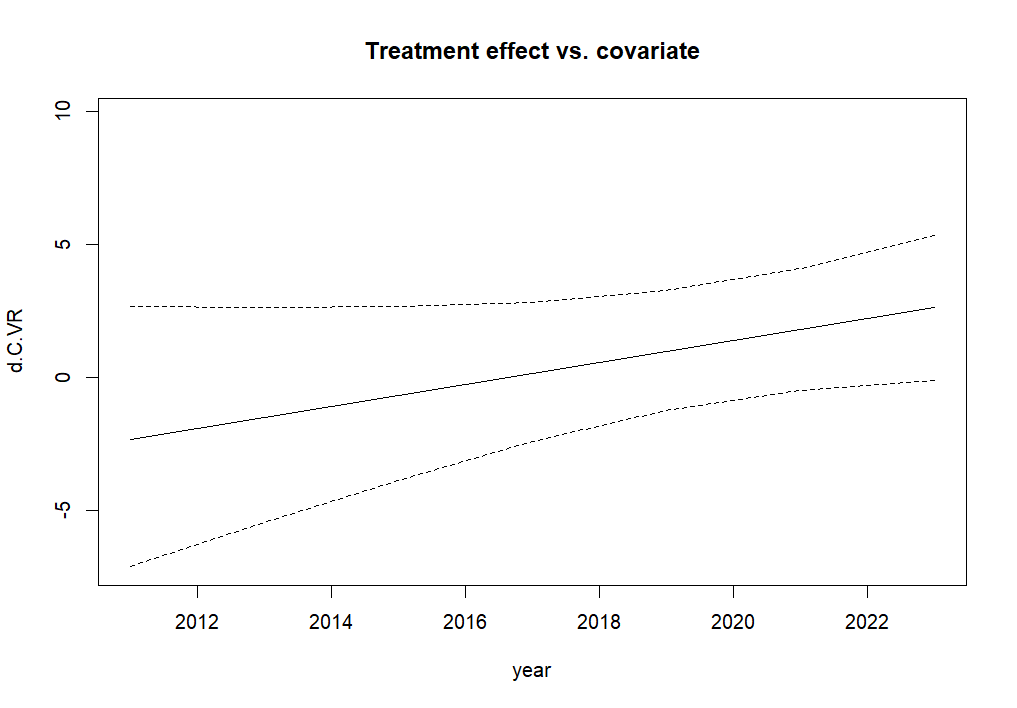

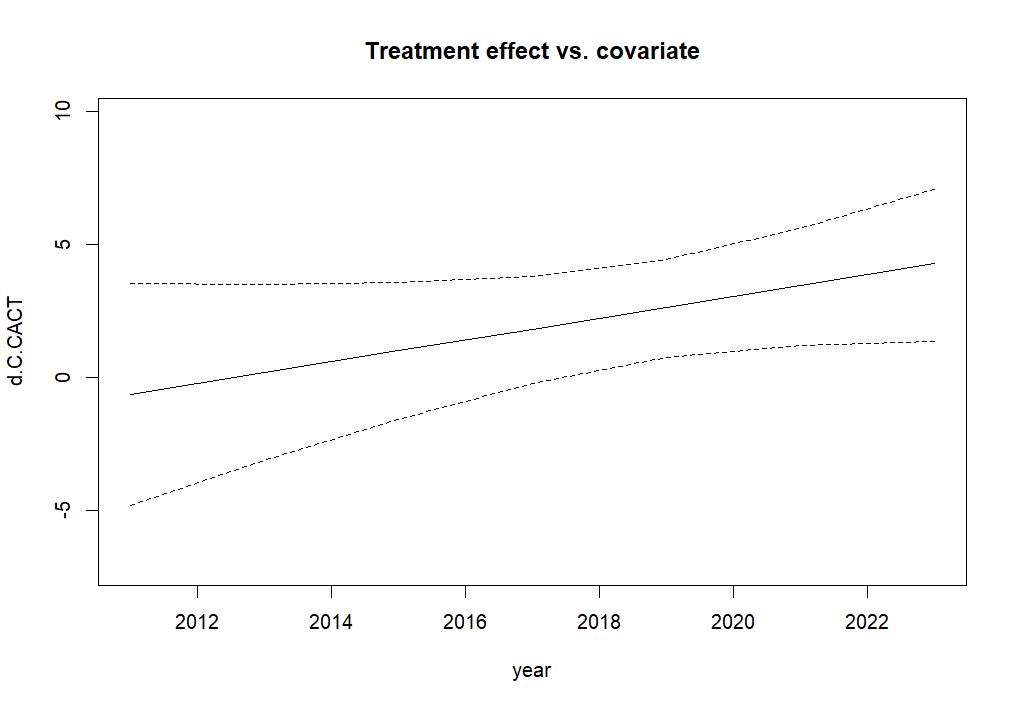

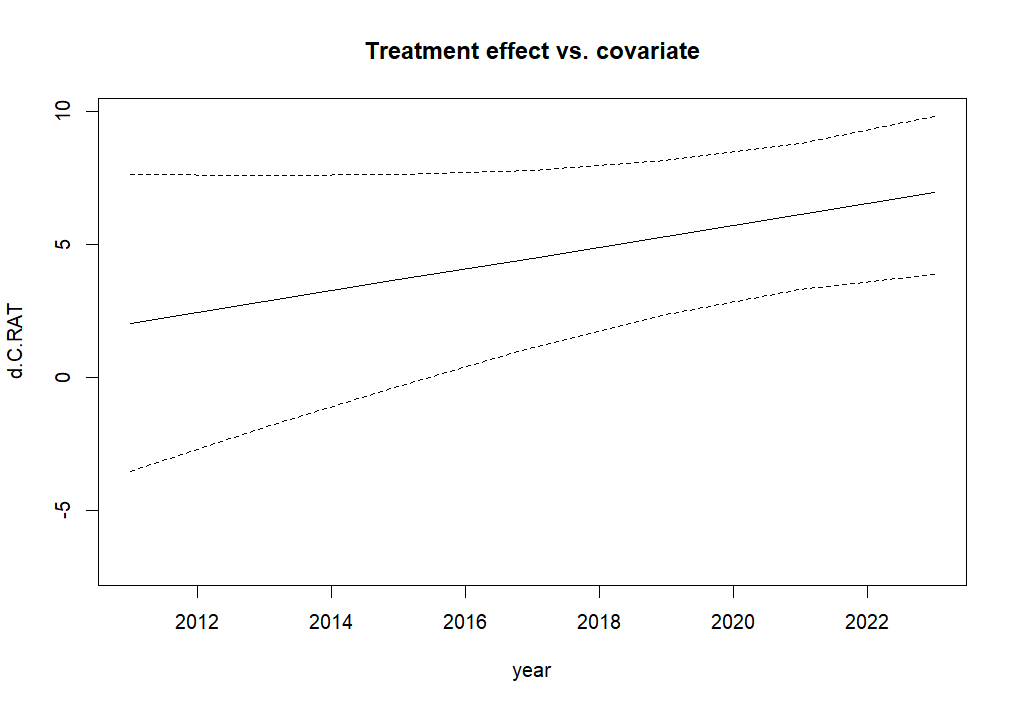

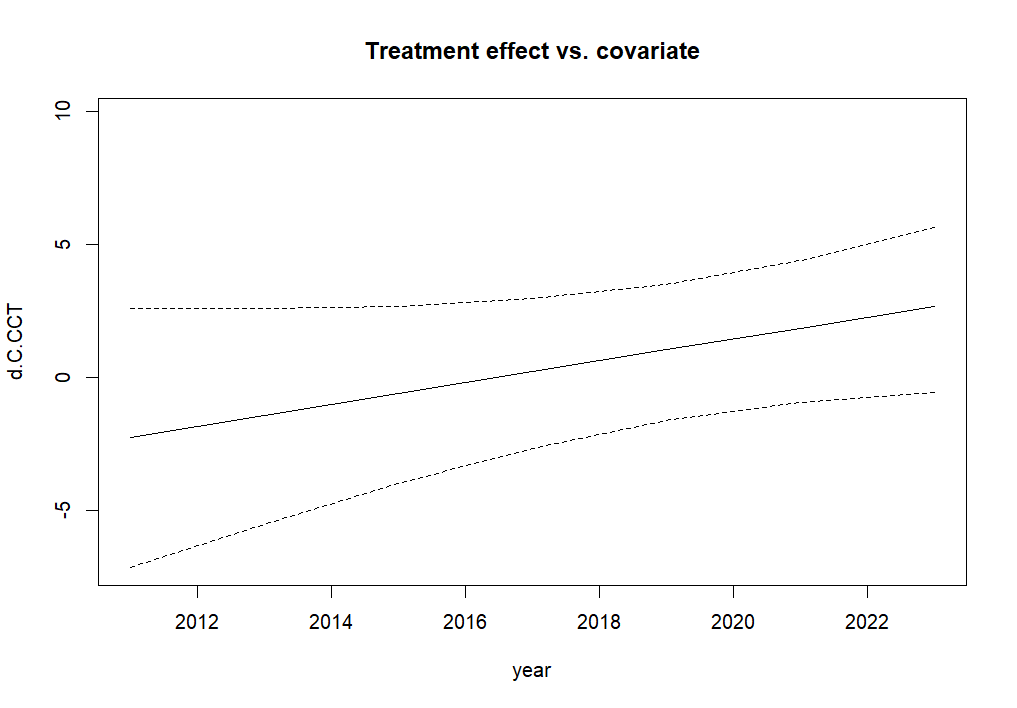


Meta-regression(Time)


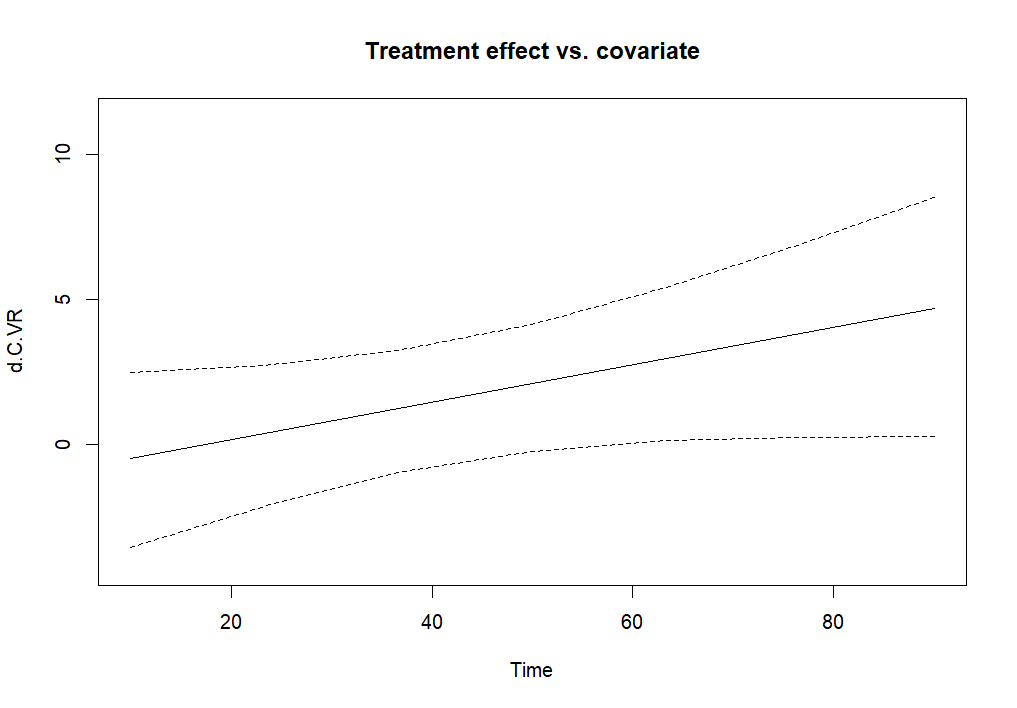

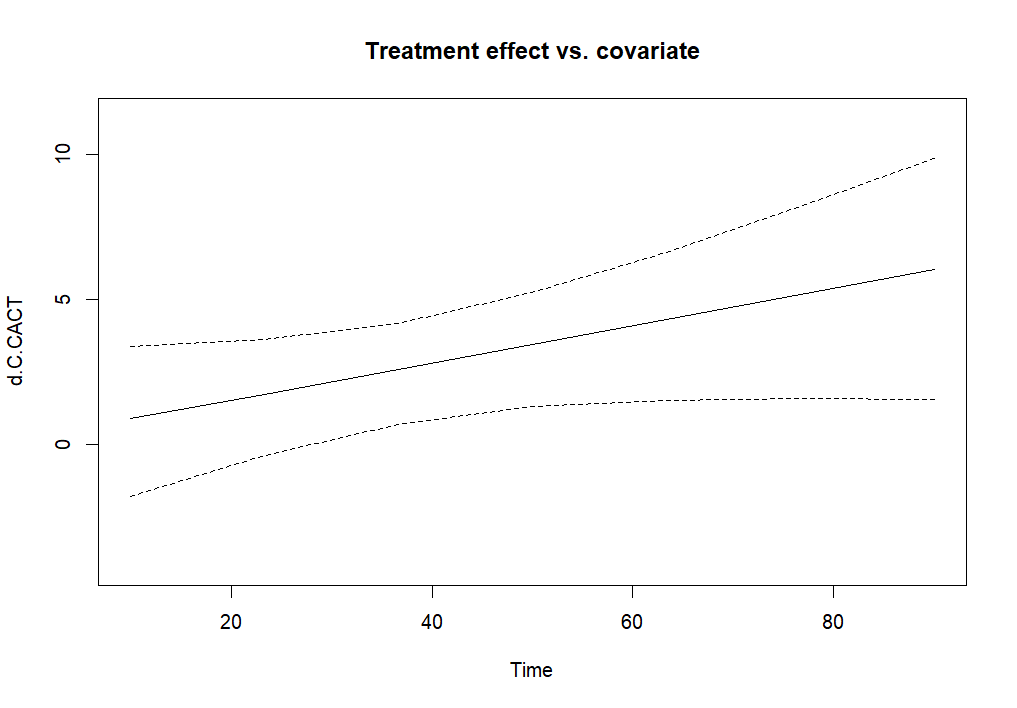

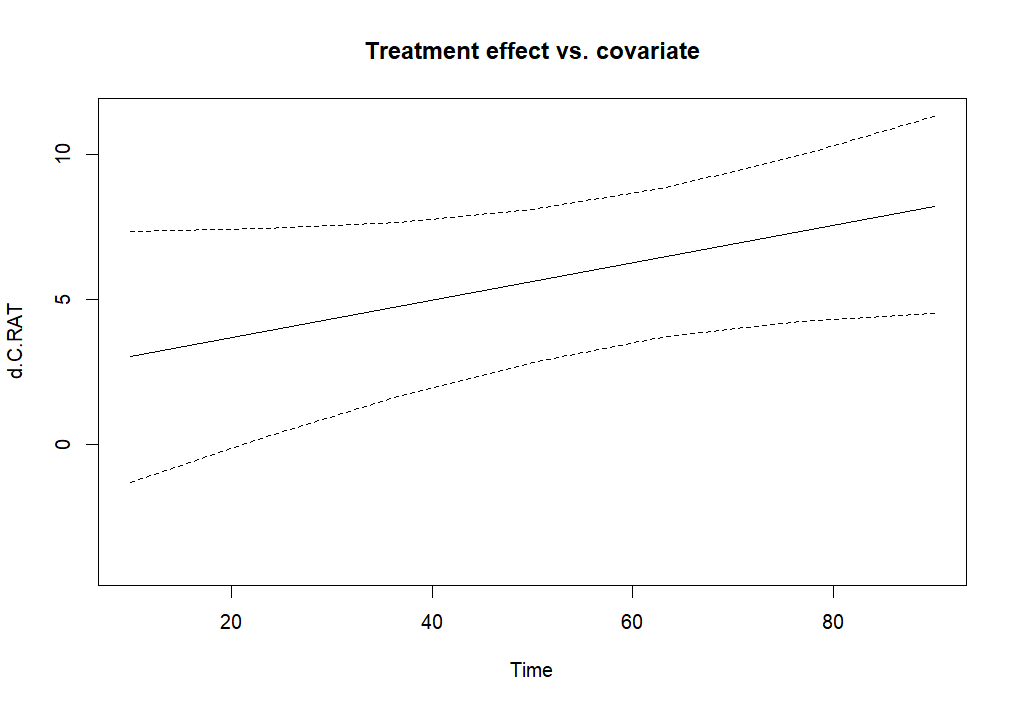

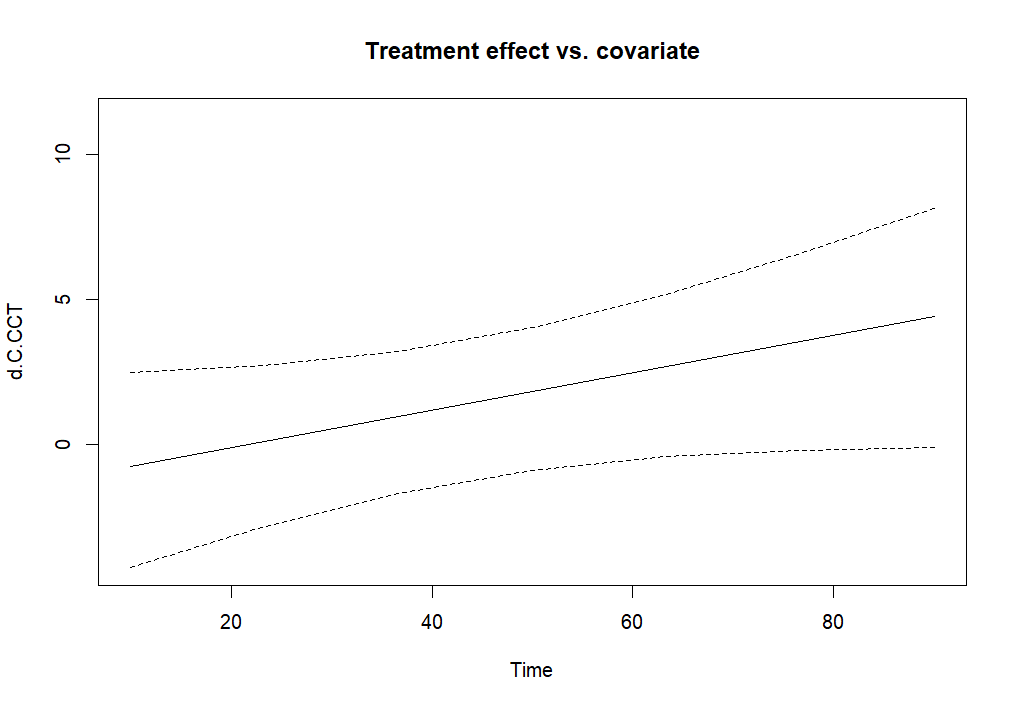

Supplement: Multimedia Appendix 5 [file jmir-v27-e73687-s005.docx]

Sensitivity Analysis(MMSE)


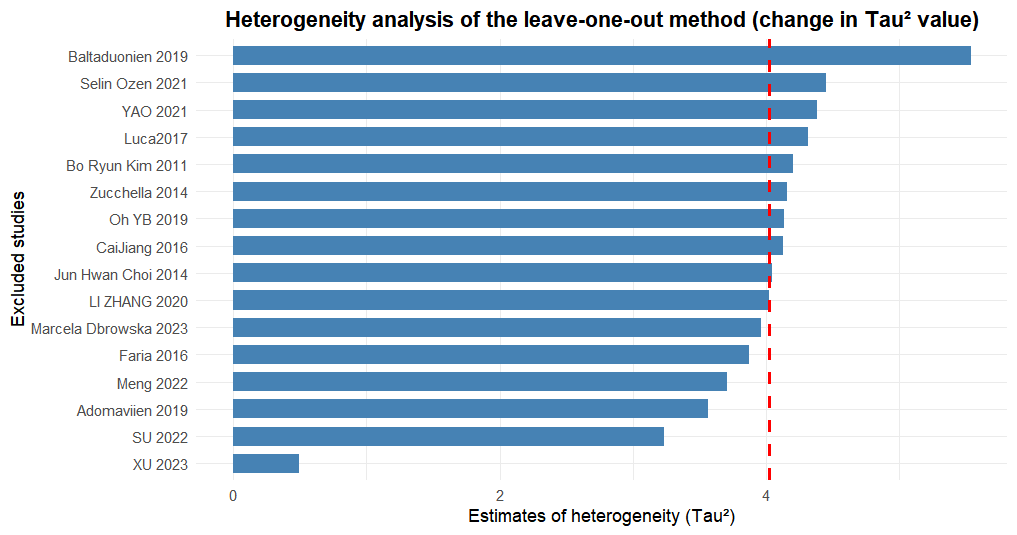

Supplement: Multimedia Appendix 6 [file jmir-v27-e73687-s006.docx]

Node-splitting analysis(MOCA)


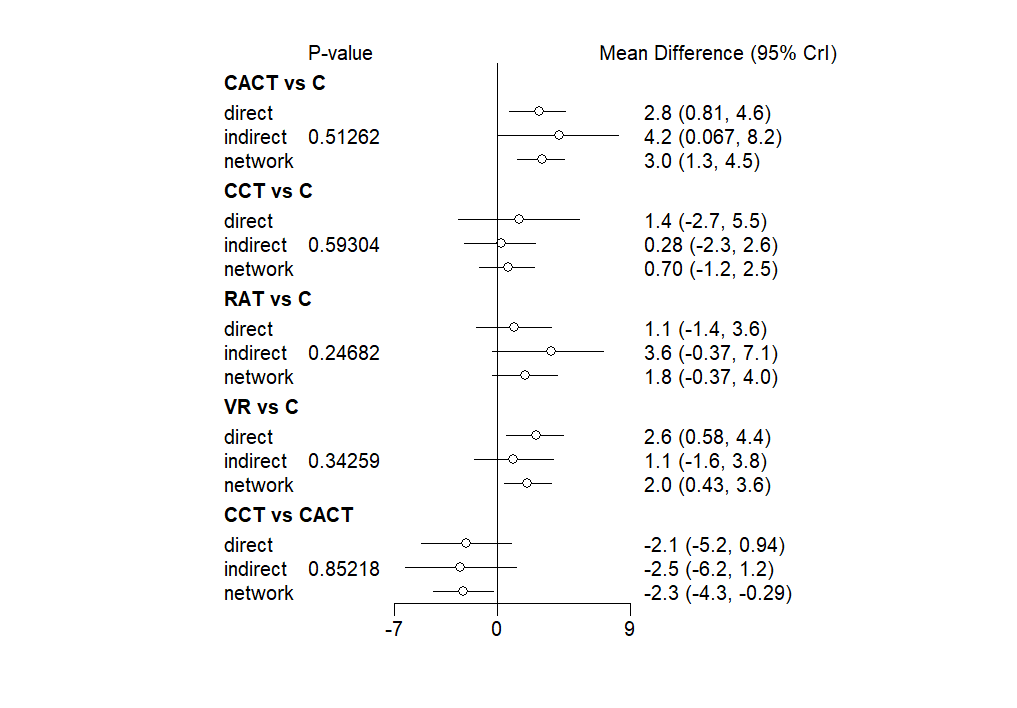

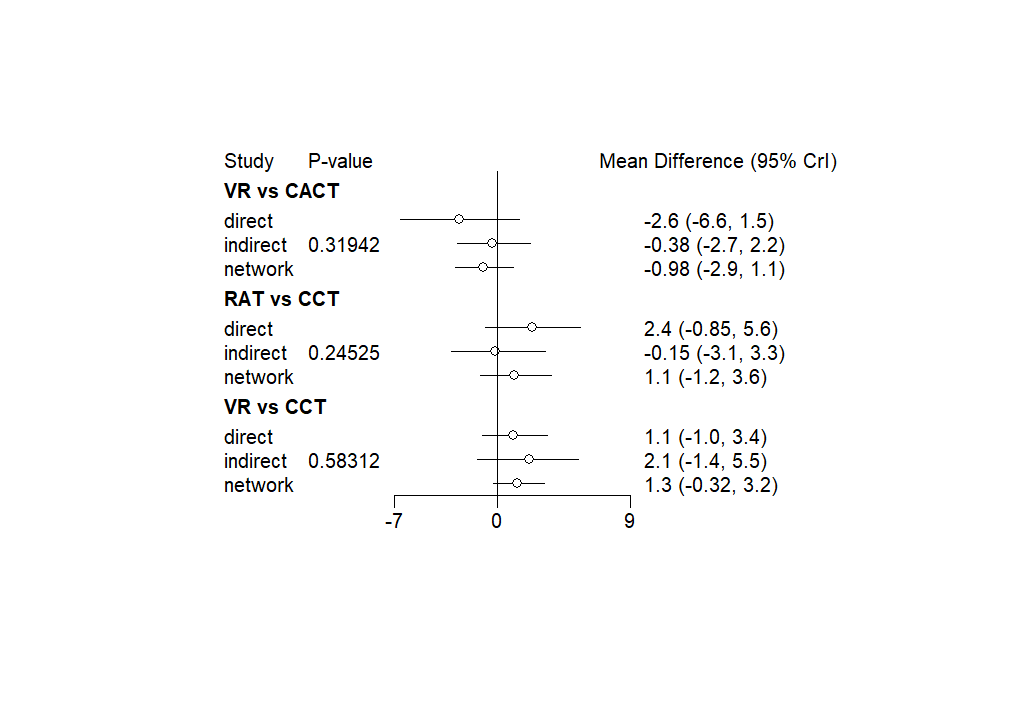


Node-splitting analysis(MMSE)


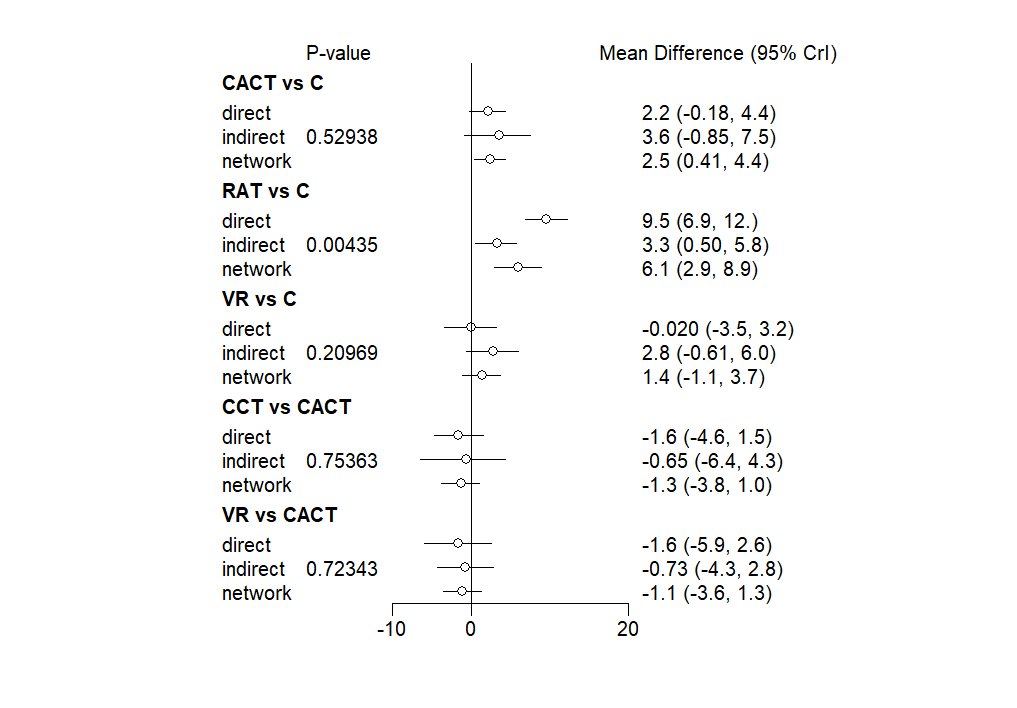

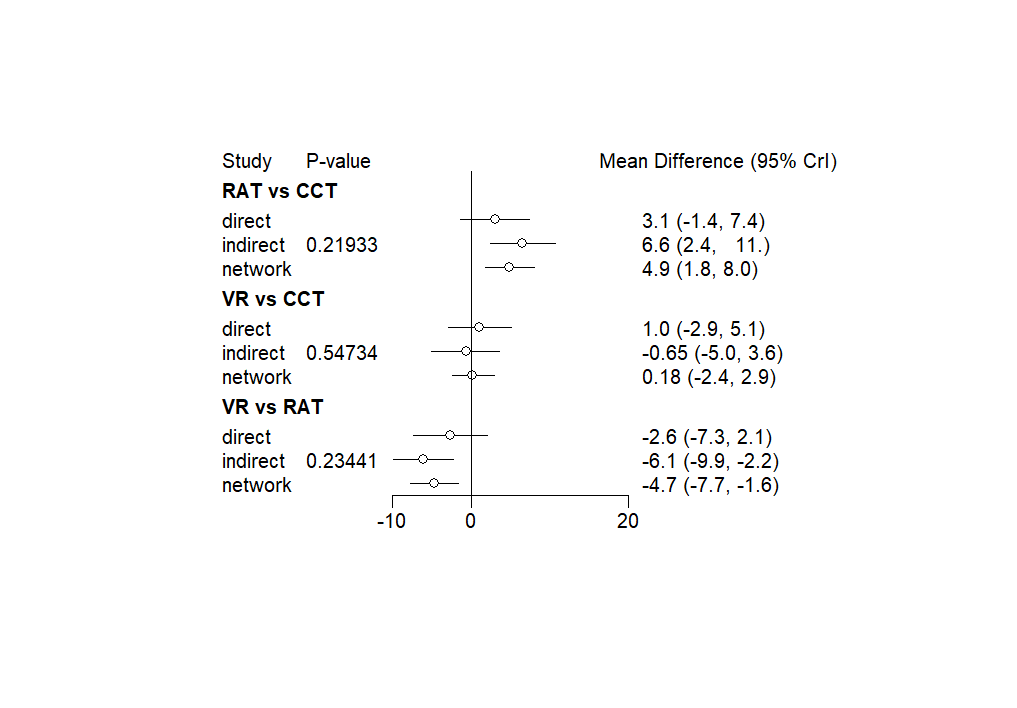

Supplement: Multimedia Appendix 7 [file jmir-v27-e73687-s007.docx]
