# Supplementary material for: Digital Interventions for Cognitive Dysfunction in Patients With Stroke: Systematic Review and Meta-Analysis
Source: J Med Internet Res. 2025 Jul 24;27:e73687. doi: 10.2196/73687 (PMC12288705; doi:10.2196/73687)

Subgroup analysis(MOCA)

Subgroup: Time


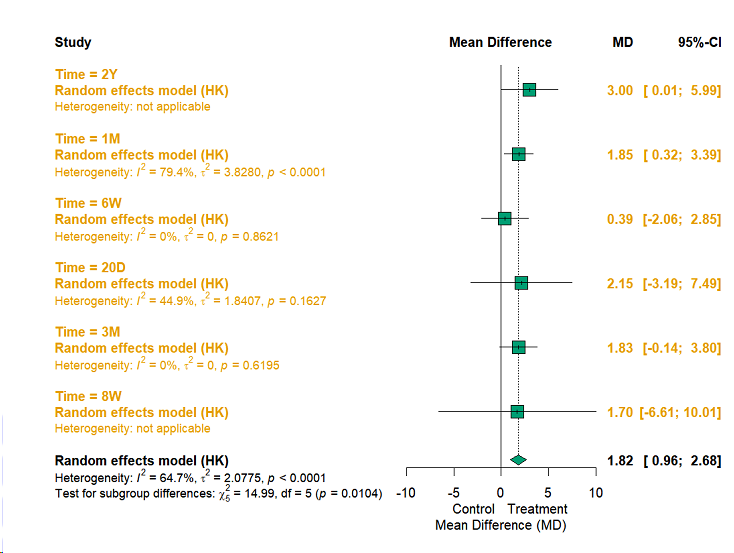


Subgroup: age


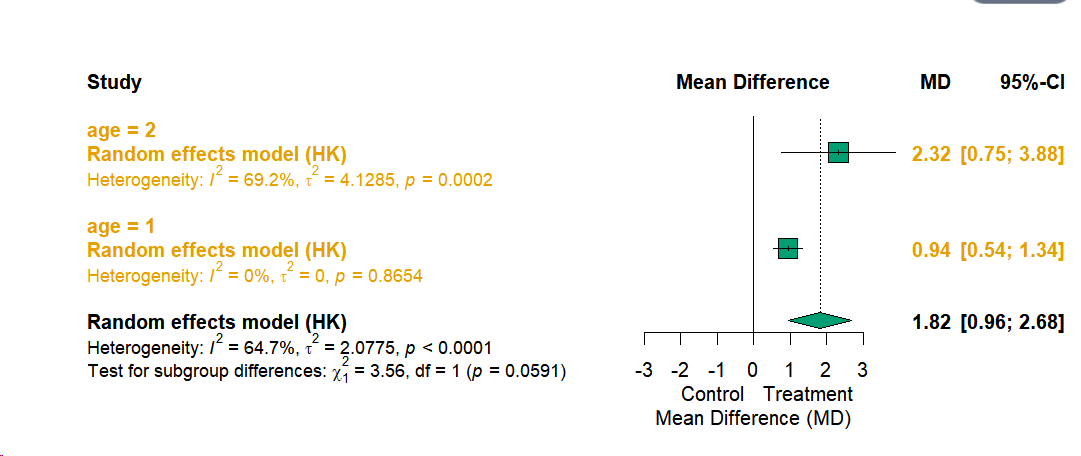


Subgroup analysis(MMSE)

Subgroup: Time


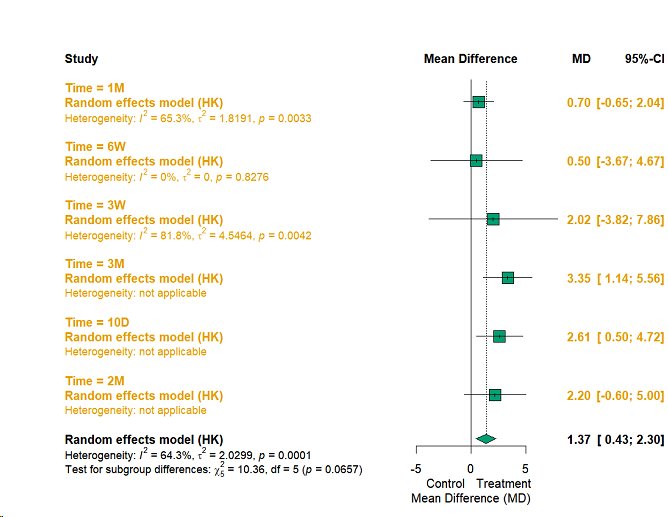


Subgroup: age


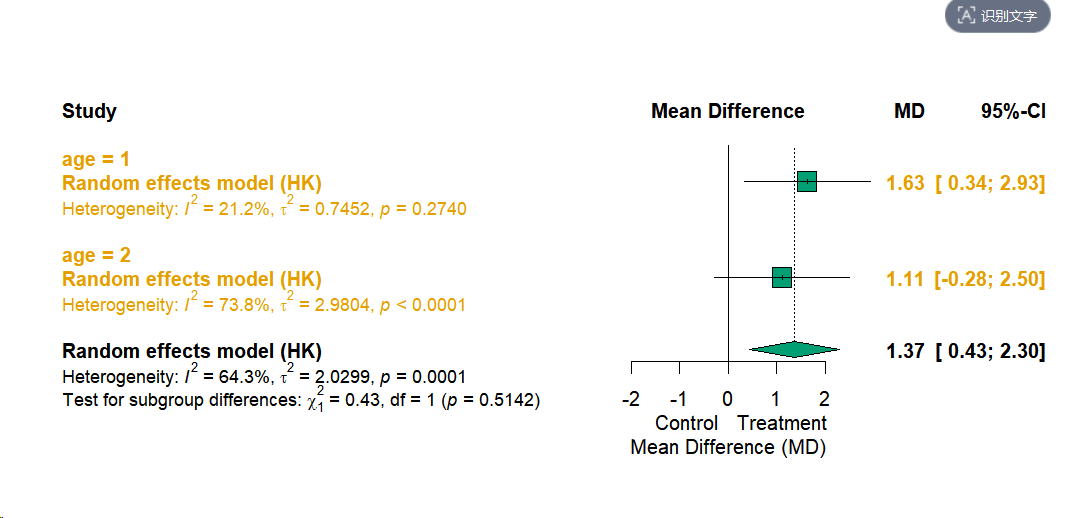

Supplement: Multimedia Appendix 4 [file jmir-v27-e73687-s004.docx]
